# Age differences in functional brain networks associated with loneliness and empathy

**DOI:** 10.1162/netn_a_00293

**Published:** 2023-06-30

**Authors:** Laetitia Mwilambwe-Tshilobo, Roni Setton, Danilo Bzdok, Gary R. Turner, R. Nathan Spreng

**Affiliations:** Montreal Neurological Institute, Department of Neurology and Neurosurgery, McGill University, Montreal, QC, Canada; Department of Psychology, Harvard University, Boston, MA, USA; Department of Biomedical Engineering, McGill University, Montreal, QC, Canada; McConnell Brain Imaging Centre, McGill University, Montreal, QC, Canada; School of Computer Science, McGill University, Montreal, QC, Canada; Mila–Quebec Artificial Intelligence Institute, Montreal, QC, Canada; Department of Psychology, York University, Toronto, ON, Canada; Departments of Psychiatry and Psychology, McGill University, Montreal, QC, Canada; Douglas Mental Health University Institute, Verdun, QC, Canada

**Keywords:** Loneliness, Empathy, Social cognition, Default network, Aging, Resting-state functional connectivity

## Abstract

Loneliness is associated with differences in resting-state functional connectivity (RSFC) within and between large-scale networks in early- and middle-aged adult cohorts. However, age-related changes in associations between sociality and brain function into late adulthood are not well understood. Here, we examined age differences in the association between two dimensions of sociality—loneliness and empathic responding—and RSFC of the cerebral cortex. Self-report measures of loneliness and empathy were inversely related across the entire sample of younger (mean age = 22.6y, *n* = 128) and older (mean age = 69.0y, *n* = 92) adults. Using multivariate analyses of multi-echo fMRI RSFC, we identified distinct functional connectivity patterns for individual and age group differences associated with loneliness and empathic responding. Loneliness in young and empathy in both age groups was related to greater visual network integration with association networks (e.g., default, fronto-parietal control). In contrast, loneliness was positively related to within- and between-network integration of association networks for older adults. These results extend our previous findings in early- and middle-aged cohorts, demonstrating that brain systems associated with loneliness, as well as empathy, differ in older age. Further, the findings suggest that these two aspects of social experience engage different neurocognitive processes across human life-span development.

## Author Summary

Feelings of loneliness emerge when a person’s desire or need for an interpersonal relationship is unmet. This state of perceived social isolation can influence social-cognitive processes that are critical for connecting with others, such as empathy. Neuroimaging studies have shown diverging functional connectivity patterns among functional brain networks between lonely younger and middle-aged adults. Here, we take a targeted approach to directly assess age-related differences in functional connectivity associated with loneliness and empathic responding in younger and older adults. We find evidence that individual differences in functional connectivity related to loneliness and empathic responding differ with age. We discuss possible mechanisms underlying these associations and their implications for brain and social functioning across the adult life-span.

## INTRODUCTION

Forming and maintaining social bonds are among the most complex of human abilities. Sociality and the emergence of social collaboration within species have been linked to larger brain sizes, with humans at the peak of this evolutionary continuum (Dunbar & Shultz, 2007). Social functioning is related to functional activation and connectivity among multiple large-scale brain systems (Mars et al., 2012; Moran et al., 2012; Mwilambwe-Tshilobo et al., 2019; Spreng et al., 2020). The importance of sociality as a determinant of brain health is most evident when social needs go unmet. Perceived social isolation, or loneliness, has a significant negative impact on mental and physical health (Cacioppo et al., 2014; Ong et al., 2016; Shankar et al., 2013; Tilvis et al., 2011). Lonely individuals experience increased risk for cognitive decline (Boss et al., 2015; Wilson et al., 2015), neuropathological burden (d’Oleire Uquillas et al., 2018; Donovan et al., 2016), and Alzheimer’s disease (Wilson et al., 2007). Although loneliness is related to adverse cognitive sequelae in age-related brain disease, much of the research investigating the impact of loneliness on brain structure and function has been conducted in younger or middle-aged adults (see Lam et al., 2021, for a review). Loneliness poses significant health risks and is a burden, particularly for older adults. However, its differential impact on brain function in early and late adulthood remains largely unexplored.

Although the experience of loneliness varies between people, it emerges because one’s need for social connection is unfulfilled (Cacioppo & Hawkley, 2009). The felt absence of connection has marked effects on the cognitive and affective processing of social signals (Bangee et al., 2014; Cacioppo et al., 2015). Loneliness influences perception and attention, resulting in negatively biased social perception and altered social functioning (Cacioppo & Hawkley, 2009). Poor perception of social cues associated with feeling lonely may hinder the ability to recognize and accurately interpret others’ thoughts and feelings, both core features of empathic responding. Preliminary evidence in younger adults indicates that this interaction may alter the impact of loneliness on the brain. Loneliness is inversely related to white matter integrity in brain regions implicated in social-cognitive processes, with higher empathy moderating this relationship (Nakagawa et al., 2015). This finding suggests that the negative behavioral association between loneliness and empathy may have a direct neural correlate, with each exerting opposing brain effects.

A growing body of neuroimaging studies now link individual differences in loneliness (Cacioppo et al., 2009a, 2009b; Düzel et al., 2019; Kong et al., 2015; Layden et al., 2017; Nakagawa et al., 2015; Wong et al., 2016) and empathy (Schurz et al., 2021a, 2021b; Völlm et al., 2006) to structural and functional changes in brain regions spanning multiple neurocognitive systems. Resting-state functional connectivity (RSFC) has demonstrated that interactions among spatially distributed brain regions underlie individual differences in loneliness (Feng et al., 2019; Mwilambwe-Tshilobo et al., 2019; Spreng et al., 2020) and empathy (Christov-Moore et al., 2020; Finn et al., 2015; Katsumi et al., 2021).

However, many open questions remain regarding the neural associations between loneliness and empathy. First, empathy is a multidimensional construct consisting of cognitive and affective components, each with distinct neural patterns (Cox et al., 2012; Schurz et al., 2021a). While behavioral evidence suggests that associations with loneliness vary in strength between components (Beadle et al., 2012), this finding may not extend to all aspects of empathic responding (i.e., empathic concern and perspective taking; Kanai et al., 2012). Second, associations between empathic responding and loneliness may change as people age. As people age, the quality of social relationships becomes more important than quantity (Carstensen, 1992). Older adults experience higher risks for loneliness (i.e., social isolation; Steptoe et al., 2013; Luhmann & Hawkley, 2016). Because loneliness impacts social perception (Cacioppo et al., 2009a, 2009b), lonely older adults may have more difficulties forming meaningful social bonds. Variability in the impact of aging on the neural correlates of cognitive and affective components of empathy (Beadle & de la Vega, 2019) may also influence age differences in the relationship between loneliness and empathic responding. Therefore, understanding the role aging plays in both behaviors may provide more insights into their individual and combined effects on the intrinsic functional connectivity of the brain.

Two studies investigating the relationship between RSFC and loneliness provide early evidence for putative age differences in brain-loneliness associations. In a large cohort of younger adults, Mwilambwe-Tshilobo et al. (2019) identified that loneliness was associated with greater RSFC between default network regions and visual and attention networks. These associations are consistent with loneliness-related neural changes in externally directed perceptual and attention networks (Schurz et al., 2021a) and support the altered social perception hypothesis of loneliness and empathy (Cacioppo et al., 2015). In contrast, a population-based study of late middle-aged adults revealed that loneliness was positively related to RSFC between the default network and fronto-parietal control and limbic regions, but not the visual network (Spreng et al., 2020). In addition, while higher default network integration was negatively correlated with loneliness in young adults (Mwilambwe-Tshilobo et al., 2019), it was positively associated with loneliness in middle-aged adults. The authors provided evidence that loneliness in middle age may precipitate more internally directed cognitive processes, mediated by the default network, as lonely individuals mentalize about desired but unmet social interactions. Combined, these two studies suggest a shift in the impact of loneliness from changes occurring in brain networks associated with externally directed cognitive processes in early adulthood to an upregulation in brain networks associated with internally directed cognitive processes in middle adulthood.

The relationship between loneliness and age is U-shaped, with peaks at 30 and 60 years of age (Luhmann & Hawkley, 2016). This relationship corresponds to the average ages in the younger and middle-aged studies described above (Mwilambwe-Tshilobo et al., 2019; Spreng et al., 2020), raising the intriguing possibility that, while the prevalence of loneliness may be similar, the social, cognitive, and neural sequelae may shift across the adult life-span. In the present study, we directly examine how individual and age differences in sociality—loneliness and empathic responding—relate to the intrinsic network architecture of the brain. We focus our analysis specifically on brain regions within six networks previously implicated in loneliness in younger and older adults (Mwilambwe-Tshilobo et al., 2019; Spreng et al., 2020; Lam et al., 2021): visual, dorsal attention, ventral attention, limbic, fronto-parietal, and default networks. We test the prediction that diverging loneliness-related RSFC patterns previously identified in young and middle-aged adults will be observed when directly comparing younger and older adults. Specifically, we hypothesize that younger adults will show a consistent pattern as Mwilambwe-Tshilobo et al. (2019) characterized by greater functional integration of the default network with visual and attention networks. In contrast, older adults will show greater functional integration of the default network with fronto-parietal and limbic networks that more closely aligns with the patterns observed in middle-aged adults (Spreng et al., 2020). Consistent with research indicating that older adults prioritize close social relationships (Carstensen, 1992) and that loneliness alters brain regions implicated in social functioning (Cacioppo et al., 2009a, 2009b; Kanai et al., 2012), we hypothesized that the RSFC patterns positively associated with loneliness would be inversely related to empathic responding and more robustly expressed in older adults than younger adults.

## METHODS

### Participants

Data from 220 participants were analyzed in the present study. Participants were part of a larger cohort (Spreng et al., 2022), where inclusion required both loneliness and empathy assessments, and two resting-state fMRI runs of data. Participants in the final sample included 128 younger (*M*_age_ = 22.6 years, *SD* = 3.3; range = 18–34; 75% female) and 92 older (*M*_age_ = 69 years, *SD* = 6.6, range = 60–89; 47% female) healthy adults recruited in Ithaca, New York, and Toronto, Canada (Table 1). All participants were right-handed, ranging from 18 to 89 years (*M* = 42, *SD* = 23.5). All participants provided informed consent following the guidelines set by the Institutional Review Board at Cornell University and York University.

**Table 1.** Descriptive data (mean and standard deviations) and inferential statistics for behavioral measures in younger and older adults

		Overall	Younger adults	Older adults	Significance
Demographics					
*n*		220	128	92	
Age, mean (*SD*)		42.0 (23.5)	22.6 (3.3)	69.0 (6.6)	
Gender, *n* (%)	F	122 (55.5)	75 (58.6)	47 (51.1)	
	M	98 (44.5)	53 (41.4)	45 (48.9)	
Education, mean (*SD*)		16.1 (2.6)	15.2 (1.8)	17.5 (2.9)	< 0.001***
Social measures					
UCLA Loneliness Scale, mean (*SD*)		39.6 (9.1)	40.6 (9.4)	38.2 (8.5)	0.06
SNI Size, mean (*SD*)		22.5 (12.5)	23.7 (12.4)	20.9 (12.4)	0.16
Instrumental support, mean (*SD*)		31.1 (7.6)	31.1 (8.7)	30.6 (6.8)	0.30
Emotional support, mean (*SD*)		33.9 (5.4)	32.8 (5.4)	34.7 (5.4)	0.02*
Friendship, mean (*SD*)		31.3 (6.3)	29.9 (6.6)	32.3 (5.9)	0.01**
Empathic functioning					
Reading the Mind in the Eyes (RMIE), mean (*SD*)		72.2 (10.0)	74.4 (9.8)	69.2 (9.7)	<0.001***
Toronto Empathy Questionnaire (TEQ), mean (*SD*)		39.0 (3.9)	39.1 (4.1)	38.8 (3.6)	0.65
IRI perspective taking (PT), mean (*SD*)		2.8 (0.6)	2.7 (0.6)	2.8 (0.6)	0.18
IRI empathic concern (EC), mean (*SD*)		3.0 (0.5)	2.9 (0.5)	3.1 (0.5)	0.003**
Personality					
Neuroticism, mean (*SD*)		2.5 (0.7)	2.7 (0.7)	2.2 (0.6)	<0.001***
Cognition					
NIH Cognitive Composite Score, mean (*SD*)		126.6 (14.4)	131.4 (14.7)	119.9 (10.9)	<0.001***

*Note*. IRI = Interpersonal Reactivity Index, SNI = Social Network Index, *SD* = standard deviation.

*p < 0.05, **p < 0.01, ***p < 0.001.

### Behavioral Measures

#### Loneliness measures.

Loneliness was measured using the Revised UCLA Loneliness Scale (UCLA-LS; Russell, 1996). The UCLA-LS is a 20-item questionnaire that measures subjective feelings of loneliness and perceived social isolation (Russell, 1996). This measure is well-established in the literature and highly reliable (Russell, 1996). One of the advantages of the UCLA-LS questionnaire is that it assesses loneliness indirectly, which diminishes potential response bias (Shiovitz-Ezra & Ayalon, 2012). For example, participants are asked to respond to statements such as “How often do you feel like there is no one you can turn to?” or “How often do you feel isolated from others.” Responses were provided on a 4-point Likert scale ranging from 1 (Never) to 3 (Always). Negatively worded items were reverse-scored. Higher scores reflect higher self-reported loneliness.

#### Aspects of empathic responding.

Empathy is not a unitary concept but a multidimensional construct that can be broken down into cognitive and affective components. The cognitive components of empathy describe processes that underlie the ability to understand and make inferences regarding another person’s mental state. The affective components of empathy describe the emotional reaction toward the observed experiences of another. While both components are conceptually distinct, recent work suggests that overlapping and unique brain activation patterns support the ability to understand how other people think and feel (Schurz et al., 2021a). To ensure that our assessment of empathic responding reflects cognitive and affective neurocognitive processes, participants completed a performance-based assessment of emotional recognition in addition to two self-report questionnaires that represent subdomains of empathy along these two dimensions:

1. The Reading the Mind in the Eyes (RMIE) task was originally conceptualized as a theory of mind questionnaire (Baron-Cohen et al., 2001). However, recent work suggests that the RMIE measures emotional recognition, not theory of mind (Oakley et al., 2016). We included the RMIE as a task-based measure in our analysis because emotional recognition is a critical aspect of empathic responding that is also predictive of prosociality (Bailey et al., 2020). The RMIE task consists of 36 photos of the eye region of adults expressing different emotional states. Participants were asked to choose one adjective from a list of four that best expresses the internal state depicted in the photo. One point was assigned for each correct response, 0 points for incorrect. Individual items were summed to give a maximum of 36, with higher scores indicating better emotional decoding.
2. The Toronto Empathy Questionnaire (TEQ) is a self-report measure that primarily assesses emotional empathy (Spreng et al., 2009). It consists of 16 items in which participants respond on a 5-point Likert scale ranging from 0 (Never) to 4 (Always). Negatively worded items were scored in reverse. Examples of items in the TEQ include “I can tell when others are sad even when they do not say anything” and “When I see someone being treated unfairly, I do not feel very much pity for them.”
3. The Interpersonal Reactivity Index (Davis, 1980) is a self-report questionnaire that consists of four subscales that assess different aspects of empathy: (1) Perspective taking (PT), the ability to take another person’s psychological point of view; (2) Fantasy, the ability to project oneself onto fictional characters; (3) Empathic Concern (EC), the tendency to experience feelings of sympathy and compassion for others; and (4) Personal Distress, a measure of the aversive response one feels when observing the negative experience of others. For this study, we only included measures of PT and EC because we were specifically interested in assessing cognitive and affective aspects of empathic responding that were other-focused (i.e., PT, EC) rather than self-focused (Fantasy, Personal Distress). Each subscale had seven items with responses made on a 5-point Likert scale ranging from 1 = “Does not describe me well” and 5 = “Describes me well”. Negatively worded items were scored in reverse.

#### Covariates.

Several demographic, social, cognitive, and personality variables associated with loneliness and aging were included as covariates in our analyses. Demographic variables included age, gender, and educational attainment. We also included the study site as a covariate since participants were part of a multisite cohort study (Spreng et al., 2022). Since our study focused on identifying age-related differences in the behavioral and neural associations between loneliness and empathy, we needed to account for known age-dependent factors that influence social and brain functioning. Age differences in loneliness are due to differences in the distribution of risk factors. For example, older adults accumulate disproportionate risk factors contributing to loneliness (Luhmann & Hawkley, 2016), two of which are poor cognitive functioning and social isolation. Evidence suggests that loneliness may accelerate cognitive decline among older adults (Shankar et al., 2013; Wong et al., 2016), and previous work highlights the need to account for the confounding effects of objective social isolation when examining loneliness among older adults (Steptoe et al., 2013). Age differences in loneliness may also be due to normative age-related changes in the quantity and quality of social relationships. Aging is marked by significant transitions in the size and composition of social relationships that lead to shrinking social network size to prioritize close social ties (Carstensen, 1992). Social relationship quantity and quality are negatively correlated with loneliness. However, having few high-quality relationships is a much stronger predictor of loneliness (Luhmann & Hawkley, 2016). Thus, accounting for the quantity and quality of social relationships may be important factors in how both age groups experience loneliness. Therefore, we controlled for objective social isolation, relationship quality, and global cognitive function measures to account for normative social network size and cognitive declines. We used the Social Network Index (Cohen et al., 1997), NIH Toolbox Emotion Battery, and Cognition Battery (https://www.nihtoolbox.org), respectively:

1. The Social Network Index is a self-report questionnaire that assesses various aspects of social engagement with 12 different types of social relationships (e.g., spouse, children, relative, friend, neighbor, coworker). Participants were asked to indicate the number of people they regularly talk to or see at least once every two weeks for each relationship type. The total number of people identified was summed to estimate social network size.
2. The NIH Toolbox Emotion Battery included three measures where participants were asked to report on their perception of social support and friendship available to them by others in their social networks (Salsman et al., 2013): (1) Instrumental Support: the subjective perception that others in their social network are available to provide advice in times of need; (2) Emotional Support: the subjective perception that people in their social network are available to listen to one’s concerns with understanding and caring; and (3) Friendship: the subjective perception that they have companions/friends available to them with which they can interact.
3. The NIH Toolbox Cognition Battery included a global composite score of overall cognition, which was automatically computed by averaging scores across seven cognitive function tests: the Picture Vocabulary Test and Oral Reading Recognition Test, Dimensional Change Card Sort Test, the Flanker Inhibitory Control and Attention Test, the Picture Sequence Memory Test, the List Sorting Working Memory Test, and the Pattern Comparison Processing Speed Test (Gershon et al., 2013). Higher scores represent better performance.

Beyond social isolation and cognition, certain personality traits may be risk factors for loneliness. Neuroticism is a personality trait that strongly positively correlates with loneliness (Abdellaoui et al., 2019). In addition, neuroticism has been associated with cognitive decline (D’Iorio et al., 2018). It mediates the relationship between loneliness and structural changes to dorsolateral prefrontal cortex (Kong et al., 2015). To account for the potential contribution of neuroticism when examining age differences in our analyses, we included neuroticism as a covariate. Participants completed The Big Five Aspect Scale (DeYoung et al., 2007), which is a 100-item self-report questionnaire that assesses facets of personality traits.

### Behavioral Data Analysis

We first conducted an independent samples *t* test to compare younger and older adults on all behavioral measures, including covariates. This allowed us to determine whether there were any age-related differences in self-reported loneliness and empathy. We then performed product-moment and partial correlations analyses to characterize the associations among all behavioral measures. Next, we examined associations between loneliness and each measure of empathic responding (RMIE, TEQ, IRI perspective taking, IRI empathic concern) in the full sample and separately within each age group. All covariates were included in partial correlation models (age, gender, site, education, neuroticism, cognitive composite score). The partial correlation analysis excluded participants with missing data on any of the covariate measures. In addition, given that a sizable portion of participants had missing data on the social network size measure (young adults: *n* = 32; older adults: *n* = 20), additional partial correlation analyses that included social network size as a covariate were conducted only in participants with complete behavioral data. Subjective measures of instrumental support, emotional support, and friendship were also included. Statistical significance was set at *p* < 0.05.

### Neuroimaging

Imaging data were acquired on a 3T GE750 Discovery series MRI scanner with a 32-channel head coil at the Cornell Magnetic Resonance Imaging Facility in Ithaca, NY, or on a 3T Siemens Tim Trio MRI scanner with a 32-channel head coil at the York University Neuroimaging Center in Toronto, Canada. Scanning protocols were closely matched across sites. Anatomical scans at Cornell were acquired using a T1-weighted volumetric magnetization prepared rapid gradient echo sequence (TR = 2,530 ms; TE = 3.4 ms; 7° flip angle; 1-mm isotropic voxels, 176 slices, 5 min 25 s) with 2× acceleration with sensitivity encoding. At York, anatomical scans were acquired using a T1-weighted volumetric magnetization prepared rapid gradient echo sequence (TR = 1,900 ms; TE = 2.52 ms; 9° flip angle; 1-mm isotropic voxels, 192 slices, 4 min 26 s) with 2× acceleration and generalized auto calibrating partially parallel acquisition (GRAPPA) encoding at an iPAT acceleration factor of 2. Two 10 min 06 s resting-state runs were acquired using a multi-echo (ME) EPI sequence at Cornell University (TR = 3,000 ms; TE_1_ = 13.7 ms, TE_2_ = 30 ms, TE_3_ = 47 ms; 83° flip angle; matrix size = 72 × 72; field of view (FOV) = 210 mm; 46 axial slices; 3-mm isotropic voxels; 204 volumes, 2.5× acceleration with sensitivity encoding) and York University (TR = 3,000 ms; TE_1_ = 14 ms, TE_2_ = 29.96 ms, TE_3_ = 45.92 ms; 83° flip angle; matrix size = 64 × 64; FOV = 216 mm; 43 axial slices; 3.4 × 3.4 × 3 mm voxels; 200 volumes, 3× acceleration and GRAPPA encoding). Participants were instructed to stay awake and lie still with their eyes open, breathing and blinking normally in the darkened scanner bay.

### Processing

Anatomical images were skull stripped using the default parameters in FSL BET (Smith, 2002). Brain-extracted anatomical and functional images were submitted to ME independent component analysis (ME-ICA; version 3.2 beta; https://github.com/ME-ICA/me-ica; Kundu et al., 2012, 2013). ME-ICA relies on the TE-dependence model of the BOLD signal to determine T2* in every voxel and separates the BOLD signal from non-BOLD sources of noise. Before TE-dependent denoising, time series data were minimally preprocessed: the first four volumes were discarded, images were computed for de-obliquing, motion correction, and anatomical-functional coregistration, and volumes were brought into spatial alignment across TEs. The T2* maps were then used for anatomical-functional coregistration. Gray matter and cerebrospinal fluid compartments are more precisely delineated by the T2* map than by raw EPI images (Speck et al., 2001; Kundu et al., 2017), which is an important consideration in aging research where enlarged ventricles and greater subarachnoid space often blur these boundaries. Volumes were then optimally combined across TEs and denoised. The outputs of interest included (1) spatial maps consisting of the BOLD components, (2) reconstructed time series containing only BOLD components, and (3) the BOLD component coefficient sets.

ME-ICA effectively removes distant-dependent motion-related artifacts in the fMRI data (Power et al., 2018). To retain all trials and maintain the same time series length across participants, we did not implement any additional denoising steps, such as scrubbing. Instead, we perform an image quality assessment on the denoised time series. In native space, we identified and excluded participants with unsuccessful coregistration, residual noise (framewise displacement (FD) > .50 mm coupled with denoised time series showing DVARS > 1; Power et al., 2012), temporal signal to noise ratio < 50, or fewer than ten retained BOLD-like components. Forty participants were excluded after the image quality assessment (younger adults: *n* = 12; older adults: *n* = 28). Age group and site differences in residual motion for included participants were assessed using FD calculated on the middle echo prior to processing. Statistical results are reported in Supporting Information Table S3.

The denoised BOLD component coefficient sets in native space, optimized for multi-echo fMRI RSFC analyses (Kundu et al., 2013), were used in subsequent steps. We refer to these as multi-echo functional connectivity (MEFC) data. Additional measures were taken to account for variation in the number of independent components from ME-ICA once connectivity matrices were estimated, as detailed below. MEFC neuroimages were mapped to a common cortical surface for each participant using FreeSurfer v6.0.1 (Fischl, 2012). To maximize alignment between intensity gradients of structural and functional data (Greve & Fischl, 2009), MEFC data were first linearly registered to the T1-weighted image by run. The inverse of this registration was used to project the T1-weighted image to native space and resample the MEFC data onto a cortical surface (fsaverage5) with trilinear volume-to-surface interpolation. This produces a cortical surface map where each vertex, or surface point, is interpolated from the voxel data. Once on the surface, runs were concatenated, and MEFC data at each vertex were normalized to zero mean and unit variance.

### Individualized RSFC Parcellation

We generated participant-specific functional connectomes to examine individual differences in functional brain network organization using the Group Prior Individual Parcellation (GPIP; Chong et al., 2017). This approach enables a more accurate estimation of participant-specific individual functional areas (Chong et al., 2017) and is more sensitive to detecting RSFC associations with behavior (e.g., Kong et al., 2021). The main advantage of this approach is that the correspondence among parcel labels is preserved across participants, while the parcel boundaries are allowed to shift based on the individual-specific functional network organization of each participant—thus providing a similar RSFC pattern that is shared across the population. Starting from an initial predefined group parcellation atlas, GPIP first refines each individual’s parcel boundaries relative to their resting-state fMRI data. Next, the concentration (inverse covariance/partial correlation) matrices from all subjects are jointly estimated using a group sparsity constraint. GPIP iterates between these two steps to continuously update the parcel labels until convergence, defined as no more than one vertex changing per parcel or 40 iterations. Compared to other group-based parcellation approaches, GPIP has been shown to improve the homogeneity of the BOLD signal within parcels and the delineation between regions of functional specialization (Chong et al., 2017).

Using this method, we used the MEFC data from each participant and parcellated the cortex into 400 functionally defined regions. We initialized all participants to a group parcellation atlas developed by Schaefer et al. (2018). Each parcel was matched to a corresponding network in the seven network parcellation by Yeo et al. (2011). In the present report, we included the visual, dorsal attention, ventral attention, limbic, fronto-parietal control, and default networks given their reliable associations with loneliness across the neuroimaging literature (Lam et al., 2021). In addition, as described in the Introduction, these networks have been associated with loneliness in younger (Mwilambwe-Tshilobo et al., 2019) and late middle-aged older adults (Spreng et al., 2020). Results for the full 7-network analysis, including the somatomotor network, are reported in supplemental material (Supporting Information Figures S1 and S2).

### Partial Least Squares Analysis

**Figure 1.**
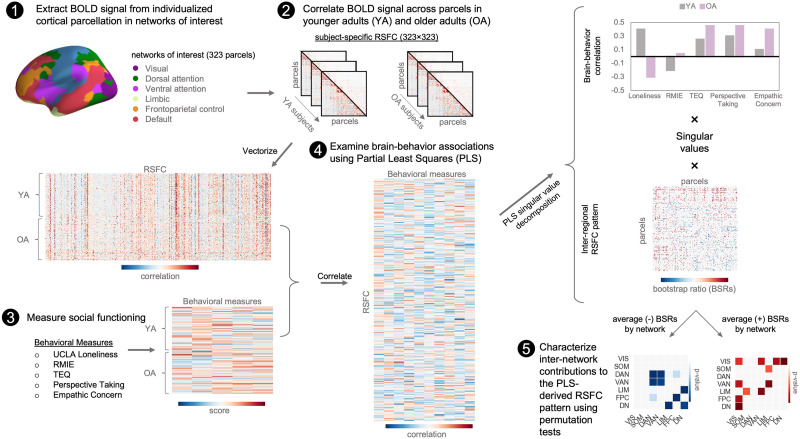
Analytic workflow of individual and age differences in functional connectivity related to loneliness and empathic responding. (1) BOLD resting-state data were extracted from subject-specific individual parcellation in six networks of interest: visual, dorsal attention, ventral attention, limbic, fronto-parietal control, and default networks. (2) Functional connectivity between parcels were constructed, forming a 323 × 323 matrix. The lower triangle of each subject’s matrix was vectorized and arranged by group assignment into a larger RSFC matrix. (3) Younger and older adults’ scores on behavioral measures of loneliness and empathic responding were combined into a matrix. (4) Partial least squares (PLS) was used to identify patterns of RSFC that maximally covary with the behavioral measures across subjects. A cross-correlation matrix generated by multiplying the RSFC and behavioral matrix was submitted to singular value decomposition. (5) Network contribution plots were used as a metric of the most reliable intra- and internetwork connections by summarizing the interregional connections from the PLS matrix.

PLS is a data-driven multivariate statistical technique used to decompose relationships between two datasets (functional connections and behavioral measures) into orthogonal sets of latent variables (LVs) that maximally covary together across participants (McIntosh & Lobaugh, 2004). The latent variables can be interpreted as optimally paired functional networks and behavioral phenotypes, respectively. We used PLS to identify age-related differences and similarities in RSFC that were directly correlated to loneliness and empathy (Figure 1).

Two datasets were constructed: a **Y** matrix containing participants’ behavioral scores on loneliness and empathy measures and an **X** matrix consisting of participants’ functional connectomes. Each row of the **Y** and **X** matrices represents the number of participants organized by group. The columns of matrix **X** correspond to the edges of the vectorized lower triangle of the RSFC matrix. The **X** and **Y** matrices were mean-centered and normalized. A correlation matrix (**R** = **X**′**Y**) was submitted to singular value decomposition (SVD) as follows:

$$R=X^{\prime }Y=USV^{\prime }$$

SVD of the cross-correlation matrix **X**′**Y** produced multiple mutually orthogonal LVs, each consisting of three elements:

1. A left singular vector (**U**) containing weights for each of the behavioral measures.
2. A right singular vector (**V**) containing weights for each of the functional connections that best characterize the relationship between RSFC among younger and older adults.
3. A scalar singular value (**S**).

Squared singular values reflect effect sizes which are proportional to the covariance between RSFC and behavior that is accounted for by each latent variable. The number of latent variables is sorted in order of proportion of covariance between the RSFC and behavior measures.

#### Participant-specific brain scores.

For each latent variable, we derived participant-specific brain scores that assess the extent to which each participant contributes to the group covariance RSFC pattern. The brain scores were calculated by multiplying the original matrix of participants’ functional connectomes (**X**) with the PLS-derived right singular vector (**V**). Partial correlations between the brain scores and each behavioral measure were conducted to account for possible confounds in the brain-behavior correlation by controlling for covariates of no interest (age, gender, site, education, neuroticism, and cognitive composite score). Covariates were partialled out of both the brain scores and behavioral measures.

#### Permutation tests.

The significance of each latent variable was assessed using permutation testing. Rows of **X** were randomly reordered and subjected to SVD iteratively, as described above. This was done 10,000 times, creating a distribution of singular values under the null distribution (McIntosh & Mišić, 2013). A *p* value was computed for each latent variable as the proportion of permuted singular values greater than or equal to the original singular value. Critically, permutation tests involve the entire multivariate pattern and are performed in a single analytic step, so correction for multiple comparisons is not required (McIntosh & Lobaugh, 2004).

#### Bootstrap resampling.

The reliability of the weights of individual RSFC connections and behavior was assessed using bootstrap resampling (Krishnan et al., 2011; McIntosh & Mišić, 2013). The brain-behavior correlations were calculated using 10,000 bootstrap samples. To identify individual connections that made a statistically significant contribution to the overall RSFC pattern, we calculated the ratio between each weight in the singular vector and its bootstrap-estimated standard error. Bootstrap ratios are equivalent to *z*-scores if the bootstrap distribution is approximately unit normal (Efron & Tibshirani, 1986). Bootstrap ratios were therefore thresholded at values of ±1.96, corresponding to the 95% CI.

#### Cross-validation.

To assess the reliability of our PLS analysis, we conducted a train-test validation of the PLS results using 5-fold cross-validation (Kebets et al., 2019). We assigned 80% of the participant data in each age group to a train set and the remaining 20% to a test set. For each fold, we used PLS to compute the RSFC (**U**_train_) and behavioral (**V**_train_) singular vectors. Then we projected the test data onto the singular vectors from the training data, allowing us to estimate participant-specific brain scores and correlation for the test set (corr(**X**_test_ **U**_train_, **Y**_test_ **V**_train_)). This was done over five folds, and the correlations between the test set original **X** (RSFC) and **Y** (behavior) matrices were performed for LV1 and LV2. The significance of the correlation was assessed using permutation tests (1,000 repetitions on the behavioral data within each group).

#### Supplementary control analyses.

We performed three additional analyses to account for possible confounding effects of the quantity and quality of social relationships, age, and motion on the primary PLS findings. First, we confirm that age group differences in the relationship between loneliness and RSFC were not due to differences in either the quantity or quality of relationships among younger and older adult participants. Two partial correlation analyses were included using social network size (quantity) and subjective measures of social support and friendship (quality). Brain-behavior correlations for the primary PLS results were computed and reported in Supporting Information Table S2B and S2C, respectively.

The second control analysis was performed to confirm that participants’ age did not influence the age differences captured in the primary PLS analysis. Age was used as a continuous variable and partialled out from the original **X** and **Y** input matrices. The two matrices were then used to run a new PLS analysis (see Supporting Information Figures S5 and S6 and Supporting Information Results 1.3). Next, we compared the covariance of each LV before and after partialling out age (Figure S7) to evaluate whether partialling out age decreased the effect size, which would be indicative of the confounding influence of age in our findings.

The last control analyses examined residual motion’s impact on RSFC in our sample. First, two independent PLS analyses were performed: (1) examining the association between RSFC and mean FD (preprocessing) and (2) identifying age differences in whole-brain RSFC (no behavior). To confirm that the RSFC pattern covarying with FD was not associated with age differences in RSFC, we correlated the brain scores derived from each PLS analysis. Relationships are plotted for the entire sample and separately for younger and older adults (Supporting Information Figure S8). Finally, to account for the effects of motion on the primary PLS analysis, mean FD post-processing was included as an additional covariate (Table S4). Results are reported in the Supporting Information.

### Network Contribution Analysis

In addition to assessing the contribution of interregional connections to the group differences, we also evaluated the extent to which network-level RSFC within and between functional networks contributed to group differences. We summarized the network contributions using the salience weights of the right singular vector (**V**). Two separate weighted adjacency matrices were constructed from positive and negative salience weights by quantifying the network-level contributions to the PLS-derived RSFC pattern. For both matrices, nodes represent parcels defined by the individual parcellation, while edges correspond to the squared salience weights of each pairwise connection. A summary of the network-level effects was estimated by assigning each parcel of the Schaefer atlas according to their respective network label based on the assignment reported by Yeo et al. (2011) and taking the average of all the squared saliences in a given network, thereby generating a 6 × 6 matrix.

In order to statistically assess the network-level effects, we took a similar approach as described above. However, we used the bootstrap ratios from the PLS-derived RSFC pattern. Here, the edges of the positive and negative adjacency matrices correspond to the thresholded bootstrap ratios of each pairwise connection of the RSFC pattern. Permutation tests were performed for statistical assessment of the pairwise networks. During each permutation, network labels for each node were randomly reordered and the mean intra- and inter- network bootstrap ratio were recalculated. This process was repeated 1,000 times to generate an empirical null sampling distribution that indicates no relationship between network assignment and RSFC pattern (Mirchi et al., 2019). The mean contribution for all intra- and internetwork network connections expressed as z-scores relative to the permuted null model are shown in Supporting Information Figure S4A–B. The significance of the pairwise connections to the network matrix was determined by estimating the proportion of times the value of the sampling distribution was greater than or equal to the original value.

## RESULTS

We measured self-reported loneliness and used self-report measures and task performance to assess cognitive and emotional aspects of empathic responding (see Table 1). We hypothesized that loneliness would be inversely related to empathic responding across the life-span (Beadle et al., 2012; Nakagawa et al., 2015). Further, we predicted that these associations might be more robust in later life as the detection of social cues declines (Moran et al., 2012; Denburg et al., 2005) and socioemotional goals become increasingly salient (Carstensen et al., 1999). Next, we examined age-related differences in the association between loneliness, aspects of empathic responding, and cortical RSFC. We acquired 20 minutes of multi-echo resting-state fMRI data (Kundu et al., 2017) and applied individualized parcellation to a subset of individuals previously examined to assess age differences in the functional architecture of the brain (Setton et al., 2023). Multivariate PLS (McIntosh & Mišić, 2013; Spreng et al., 2020; Schurz et al., 2021b) was used to identify patterns of RSFC related to individual differences in loneliness and empathic responding, as well as differences between younger and older adult age groups. Based on our previous findings from independent samples of young (Human Connectome Project; Mwilambwe-Tshilobo et al., 2019) and late middle-aged adults (UK Biobank; Spreng et al., 2020), we predicted robust age differences in the association between loneliness, aspects of empathic responding, and RSFC. Specifically, we hypothesized that age differences would arise within the default network and default network interactions with other association networks implicated in internally directed cognitive processes (Andrews-Hanna et al., 2014).

### Demographics and Descriptive Statistics

To examine whether the relationship between loneliness and empathic responding showed similar associations as prior studies (Beadle et al., 2012; Nakagawa et al., 2015), we first characterized individual and age-related differences in loneliness and subdomains of empathy within our cohort. The behavioral measures included self-reported loneliness, emotional recognition accuracy on the RMIE task, self-reported empathy, perspective taking, and empathic concern. Additionally, we controlled for nuisance or confounding variables, including scanning site, gender, education, social network size, instrumental support, emotional support, friendship, neuroticism, and global cognition (see Methods for full rationale). Table 1 summarizes the means and standard deviations of loneliness and empathic responding measures, along with all covariates included in subsequent analyses.

**Figure 2.**
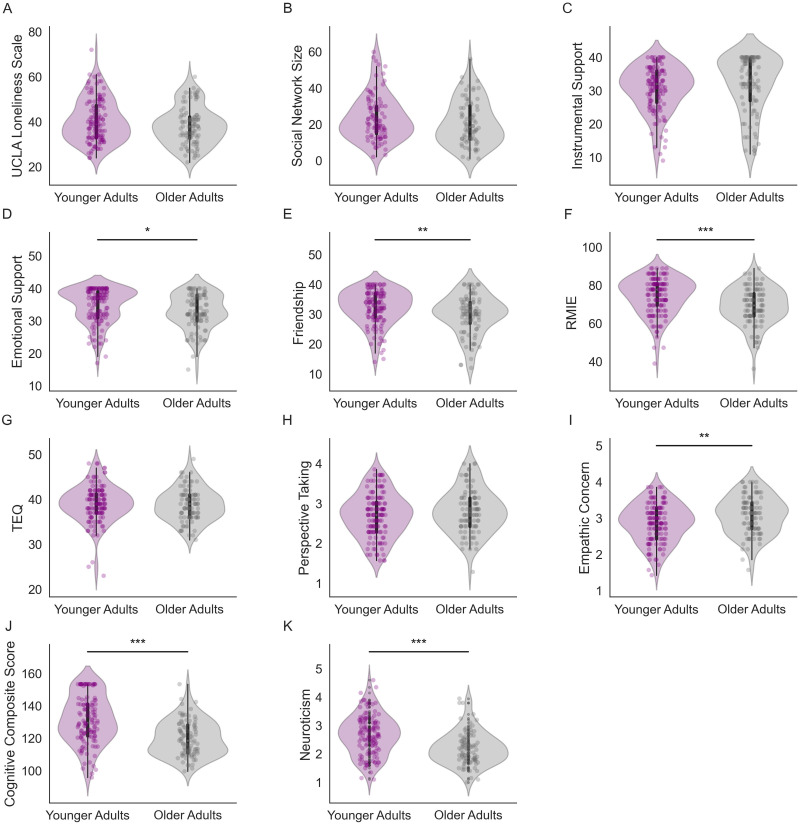
Group comparison on behavioral measures. Violin plots showing the distribution of behavioral scores in younger and older adults for (A) loneliness, (B) social network size, (C–D) social support, (E) friendship, (F–I) empathic responding measures, (J) global cognitive function, (K) and neuroticism. For comparisons on behavioral measures of interest (loneliness and empathic responding), although self-reported loneliness was similar among age groups, significant age-related differences can be observed for task-based performance of emotional recognition and in self-reported empathic concern. RMIE = Reading the Mind in the Eyes Task; TEQ = Toronto Empathy Questionnaire; **p < 0.01. ***p < 0.001.

Violin plots illustrating age differences on behavioral measures in each age group are shown in Figure 2. Scores on the UCLA loneliness scale trended lower for older, compared to younger adults (*t*(218) = 1.88, *p* = 0.06; Cohen’s *d* = 0.26; Figure 2A). Younger and older adults significantly differed on some, but not all, measures of empathy. Older adults were less accurate at emotional recognition of others’ facial expressions based on performance on the RMIE (*t*(218) = 3.94, *p* < 0.0001; Cohen’s *d* = 0.54; Figure 2F). Older adults reported greater empathic concern than younger adults (*t*(218) = −3.00, *p* = 0.003; Cohen’s *d* = 0.41; Figure 2I). No significant age differences were found on other measures of empathic functioning (TEQ: *t*(218) = 0.45, *p* = 0.65; Cohen’s *d* = 0.06; Perspective Taking: *t*(218) = −1.35, *p* = 0.18; Cohen’s *d* = 0.19; Figure 2G and 2H).

Neuroticism and normative cognition declines may influence the relationship between loneliness, empathic responding, and RSFC. Therefore, we assessed age-related differences in neuroticism and global cognition based on the NIH cognitive composite score. We also assessed whether younger and older adults differed across covariates incorporated in subsequent analyses. Older adults scored lower on neuroticism (*t*(207.79) = 5.32, *p* < 0.001; Cohen’s *d* = 0.72; Figure 2K) and had lower overall cognitive function (*t*(210) = 6.2, *p* < 0.001; Cohen’s *d* = 0.86; Figure 2J). Social networks tend to shrink with age, and evidence from longitudinal work has found that objective social isolation may confound the effects of loneliness among older adults (Steptoe et al., 2013). To determine whether such differences were present within our sample, we compared the network sizes between younger and older adults and found no age difference (*t*(166) = 1.42, *p* = 0.16; Cohen’s *d* = 0.22; Figure 2B). We also included measures of perceived social support and friendship to assess the quality of social relationships participants felt they had access to. Older adults reported greater perceived emotional support (*t*(215) = 2.45, *p* = 0.02; Cohen’s *d* = 0.34; Figure 2D) and friendship (*t*(215) = 2.83, *p* = 0.01; Cohen’s *d* = 0.39; Figure 2E), but no significant differences were found for instrumental support (*t*(215) = −1.08, *p* = 0.28; Cohen’s *d* = 0.15; Figure 2C).

Next, we assessed the association among all behavioral variables across the full sample (see Table 2). Scores on the UCLA loneliness scale correlated negatively with perspective taking, empathic concern, social network size, instrumental support, emotional support, and friendship. Scores on the UCLA loneliness scale were positively associated with neuroticism and cognitive function. Accuracy on the RMIE was not significantly associated with loneliness or other empathy subdomain measures. RMIE accuracy in this sample was positively correlated with emotional support and cognitive function. The TEQ was correlated with perspective taking and empathic concern subscales of the IRI and with participant social network size. Empathic concern was positively correlated with perspective taking, emotional support, and friendship, and negatively associated with neuroticism and cognitive function. Social network size was positively correlated with friendship. Emotional support was positively correlated with friendship. Neuroticism was negatively correlated with emotional support and friendship, and contrary to expectation, positively correlated with global cognitive function.

**Table 2.** Correlation of measures across all participants

	1	2	3	4	5	6	7	8	9	10	11	12
1. Loneliness	–											
2. RMIE	0.09	–										
3. TEQ	−0.12	0.09	–									
4. IRI Perspective Taking	−0.17*	0.03	0.22***	–								
5. IRI Empathic Concern	−0.17*	−0.02	0.41****	0.42****	–							
6. Social network size	−0.21**	0.02	0.24**	0.1	0.17*	–						
7. Instrumental support	−0.34****	0	0.04	0.02	0.04	−0.03	–					
8. Emotional support	−0.57****	0.15*	0.09	0.19**	0.06	0.01	0.44****	–				
9. Friendship	−0.68****	0.03	0.08	0.17*	0.06	0.27***	0.30****	0.59****	–			
10. Neuroticism	0.45****	0.13	0.04	−0.33****	0.18**	0.03	−0.09	−0.25***	−0.23***	–		
11. Education	−0.12	−0.05	0	0.03	0.13	−0.03	0.19**	−0.01	−0.11	−0.1	–	
12. Cognition composite score	0.14*	0.42****	0.01	−0.02	−0.17*	0.07	−0.12	−0.08	−0.05	0.27****	−0.07	–

*Notes*. Correlation values in **boldface** are statistically significant. RMIE = Reading the Mind in the Eyes Task; TEQ = Toronto Empathy Questionnaire. Social network size, neuroticism, education, and cognition composite scores are included as covariates in analyses.

*p < 0.05, **p < 0.01, ***p < 0.001.

### Behavioral Associations Between Loneliness and Empathic Responding in Younger and Older Adults

Our previous results showed significant age differences on some aspects of empathic responding. We therefore examined the association between loneliness and empathy measures in both younger and older adults separately (controlling for gender, site, education, neuroticism, and global cognitive function). The partial correlations between the gold standard UCLA loneliness scale and the four measures of empathic responding are shown in Supporting Information Table S1 for both age groups. In younger adults, loneliness was not significantly correlated with any empathic responding measures. In contrast, loneliness in older adults was significantly and negatively associated with TEQ, perspective taking and empathic concern, but not accuracy on the RMIE task. Two additional partial correlation correlations were performed to assess the influence of social network quantity and quality on the behavioral associations observed in younger and older adults (Table S1B). Although not all participants in the cohort completed the objective social network size measure, we reanalyzed the associations between loneliness and empathic responding by including social network size as a covariate and found that no significant associations remained (Table S1C). However, when we included instrumental support, emotional support, and friendship as proxy measures of social relationship quality, loneliness across the full sample was significantly correlated with accuracy on the RMIE while all remaining measures of empathic functioning were no longer significant (Table S1D).

### RSFC Associations With Loneliness and Empathic Responding in Younger and Older Adults

Next, we implemented a data-driven multivariate approach to identify patterns of RSFC related to loneliness and empathic responding in younger and older adults (Figure 1). RSFC was examined among the visual, dorsal attention, ventral attention, limbic, fronto-parietal, and default networks. Two significant LVs capturing distinct RSFC patterns reflecting age-related differences and similarities in social behavior were observed. A scree plot showing the covariance explained for all LVs is shown in Supporting Information Figure S3. Detailed results examining the impact of social relationship quantity and quality on brain-behavior associations identified by each LV are provided in Supporting Information Results 1.1.

#### Age differences in RSFC related to loneliness.

**Figure 3.**
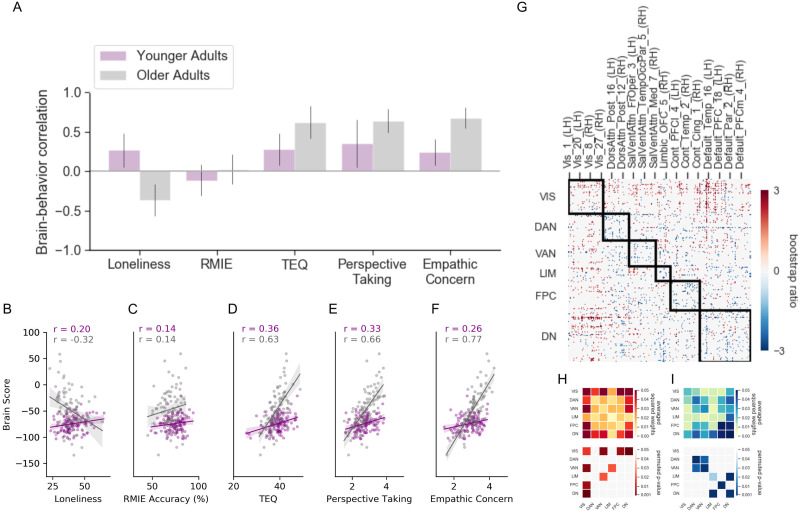
PLS analysis of brain-behavior covariance for LV1. (A) Correlation between behavioral loneliness, empathic responding measures, and RSFC in younger and older adults. Error bars show 95% confidence intervals determined by bootstrap resampling. (B–F) Scatterplots show correlations between participant brain scores and behavioral measures controlling for age, site, gender, education, neuroticism, and cognition as a function of each behavioral measure. (G) Correlation matrix of the reliable pairwise functional connections associated with behavior. The matrix bootstrap ratios are thresholded at ±2 to 3. Network-level contributions to the positive (H) and negative (I) connectivity pattern for LV1: top matrices show the averaged squared salience weights, which reflects a summary of the connectivity pattern; bottom matrices show significant network contribution estimated using permutation testing on the correlation matrix in panel G. Behaviors that correlate positively with the pattern are represented in warm colors, and negative brain-behavior correlations in cool colors. VIS = Visual network; DAN = Dorsal attention network; VAN = Ventral attention network; LIM = Limbic network; FPC = Frontoparietal control network; DN = Default network.

The first LV revealed a pattern of RSFC that dissociated younger and older adult RSFC associated with loneliness (*p =* 0.04; 26.02% covariance explained; Figure 3). Additionally, self-reported empathy covaried in both groups with a pattern of RSFC observed for loneliness in young adults. No reliable relationship between emotional recognition on the RMIE task and RSFC was found in either age group. To assess the specificity of the brain-behavior correlations in each group, we performed partial correlation analyses controlling for the effects of gender, site, neuroticism, and cognitive function on participants’ brain score. Figure 3B–3F depicts scatterplots of the relationship between participant brains scores, representing the weighted values of the RSFC pattern of the LV controlling for covariates and all five behavioral measures. Results indicate that significant brain-behavior correlations for LV1 were robust, as they remained significant after controlling for covariates in both age groups (see Supporting Information Table S2A for statistical results).

Next, we summarized the average connectivity pattern within and between networks. We examined the significance of the pairwise connections using permutation testing (Figure 3H and 3I). The most notable feature that emerged was a dissociation between the connectivity of the visual network and heteromodal association regions. This dissociation reflects the age interaction in loneliness on RSFC. In younger adults, higher loneliness was associated with greater visual network connectivity with ventral attention, fronto-parietal control, and default networks (Figure 3H). This pattern of RSFC was also associated with self-reported empathy (TEQ, perspective taking, and empathy) in both age groups. In contrast, higher loneliness in older adults was associated with more intranetwork RSFC of attention, fronto-parietal, and default networks, and greater RSFC between default and fronto-parietal, limbic, and dorsal attention networks (Figure 3I).

#### RSFC related to subdomains of empathic responding.

**Figure 4.**
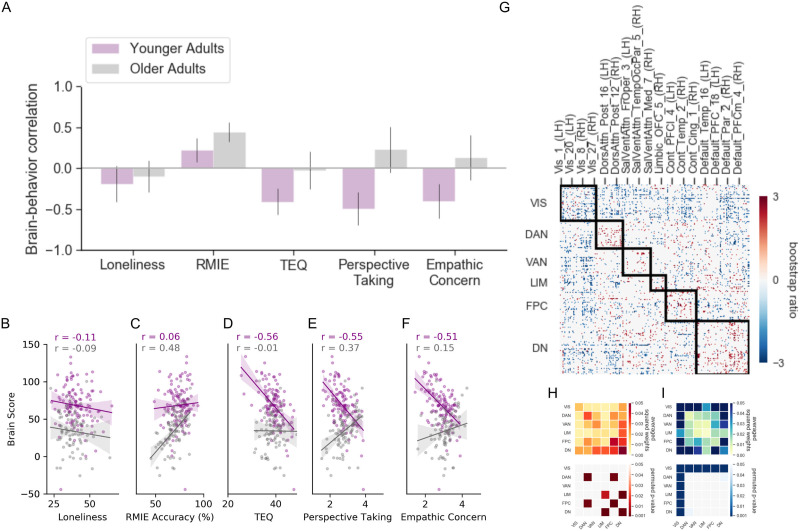
PLS analysis of brain-behavior covariance for LV2. (A) Correlation between behavioral loneliness, empathic responding, and RSFC in younger and older adults. Error bars show 95% confidence intervals determined by bootstrap through bootstrap resampling. (B–F) Scatterplots show correlations between participant brain scores and behavioral measures controlling for age, site, gender, education, neuroticism, and cognition as a function of each behavioral measure. (G) Correlation matrix of the reliable pairwise functional connections associated with behavior. The matrix bootstrap ratios are thresholded at ±2 to 3. Network-level contributions to the positive (H) and negative (I) connectivity pattern for LV1: top matrices show the averaged squared salience weights, which reflects a summary of the connectivity pattern; bottom matrices show significant network contribution estimated using permutation testing on the correlation matrix in panel G. Behaviors that correlate positively with the pattern are represented in warm colors and negative brain-behavior correlations in cool colors.

A second significant pattern revealed shared and diverging associations related to various facets of empathic responding (*p* < 0.01; 16.53% covariance explained; Figure 4). The brain-behavioral correlations for both groups are shown in Figure 4A. Across both age groups, better performance on emotional recognition on the RMIE task correlated positively with greater intra- and internetwork RSFC among regions in heteromodal association cortex (Figure 4G). This pattern was particularly prominent within the dorsal attention, fronto-parietal, and default networks, and between default to other heteromodal networks and fronto-parietal to dorsal attention networks (Figure 4H). LV2 also captured a RSFC pattern of age group differences in the relationship between RSFC and all empathic responding measures. Younger adults with higher scores on the TEQ, perspective taking, and empathic concern showed strong intranetwork connectivity of the visual network and connectivity between the visual network with the other five networks (Figure 4I). No significant associations between RSFC and these three self-report measures of empathic responding were found in older adults. When controlling for covariates of no interest, significant brain-behavior correlations remained in younger adults for TEQ, perspective taking, and empathic concern (Supporting Information Table S2A). For older adults, a significant positive brain-behavior correlation emerged for perspective taking. Scatterplots of the relationship between the corrected brain scores and each behavioral measure can be found in Figure 4B–4F.

### Cross-Validation of PLS Results

To account for potential overfitting from our PLS analysis, we conducted a second analysis to assess the stability of the identified patterns (see Methods for more details). A fivefold cross-validation was performed on the two LVs by correlating the RSFC-behavior associations of each LV in the training set and calculating the mean correlation across folds. The mean correlation was strongly correlated (*r* = 0.54). RSFC-behavior correlations in the test set representing 20% of the sample for the test set were lower but remained significantly correlated across fold (*r* = 0.19; *p* = 0.003), suggesting that PLS LVs estimated from train data were stable in the testing set.

## DISCUSSION

We examined the relationship between loneliness, empathic responding, and RSFC in younger and older adults to identify age group differences in the associations between sociality and brain function. Older adults reported feeling less lonely and more empathic, yet scored lower on a performance-based measure of emotional recognition (RMIE). Negative associations between loneliness and empathy were observed across the life-span, with more robust associations detected for older versus younger adults. Predicted age differences were observed in the association between loneliness, empathic responding, and RSFC. Brain and behavioral associations did not differ for loneliness and empathy in younger adults. Positive associations were observed for both aspects of sociality and RSFC between visual regions and spatially distributed brain systems. Older adults showed a divergence in RSFC associations between loneliness and empathy. Higher self-reported loneliness in older adults was associated with greater RSFC within heteromodal association networks and between attention (dorsal and ventral) and the default and fronto-parietal and limbic networks. In contrast, and consistent with younger adults, higher self-reported empathy for older adults was associated with greater visual network connectivity to the cortex. These findings adjudicate previous reports (Mwilambwe-Tshilobo et al., 2019; Spreng et al., 2020) and reveal that sociality and RSFC associations differ for young and older adults. Our findings also show that age differences are specific to loneliness and involve cortical association networks related to internally directed cognition and socioemotional processing.

### Age Differences in RSFC Related to Loneliness

We observed a difference in the relationship between RSFC and loneliness between younger and older adults. Integration of visual and association networks was related to higher loneliness in young. In contrast, higher loneliness in older adults was marked by lower RSFC of visual regions and greater intra- and internetwork RSFC among higher order association networks. These findings support our hypothesis that age-related differences in the association between loneliness and brain function reflect a shift from externally to internally oriented processing regions, reconciling previous reports (Mwilambwe-Tshilobo et al., 2019; Spreng et al., 2020). Importantly, no shared pattern relating loneliness to RSFC was observed between age groups, suggesting a qualitatively different pattern in the neural basis of loneliness across the life-span. Although we could not test this directly, we suggest that these differences reflect a shift in the perception and experience of loneliness into older age.

In younger adults, integration of visual and association networks may reflect increased social perception demands to monitor for threatening social cues or seek new opportunities for social connection (Cacioppo & Hawkley, 2009). In contrast, for older adults, functional segregation of the visual network and increased integration within and between higher order association networks related to loneliness may reflect a shift toward more internally directed processing (cf. Spreng et al., 2020), consistent with an age-related shift toward prioritizing socioemotional goals. Instead of searching for new social contacts, older adults have smaller social networks that prioritize close social connections (Carstensen, 1992). As the pursuit of new social experiences declines with age, lonely older adults may rely more on reminiscing about past experiences (Ross & Inagaki, 2023) or mentalizing about future social engagements (Spreng et al., 2020). Autobiographical recollection and future thinking are robustly related to the default network and its interactions with other association networks (Andrews-Hanna et al., 2014; Schacter et al., 2012), which closely converges with the connectivity pattern associated with loneliness in older but not younger adults in our study. In the context of previous reports (Mwilambwe-Tshilobo et al., 2019; Spreng et al., 2020), our findings suggest that the experience of loneliness shifts over the adult life-span. However, given that our study was cross-sectional, an alternative explanation for the age differences found could be due to older adults experiencing loneliness more chronically than younger adults. Future studies are necessary to examine the experience of loneliness and associated cognitive, social, and neural antecedents and sequelae into older age (Bzdok & Dunbar, 2020, 2022; Spreng & Bzdok, 2021).

Another possible explanation for the age differences in RSFC associated with loneliness is that healthy aging is characterized by brain network dedifferentiation (Chan et al., 2014; Malagurski et al., 2020; Setton et al., 2023). Dedifferentiation in older adulthood may, in part, compensate for the functional reorganization of the aging brain (Reuter-Lorenz & Cappell, 2008), although some aspects of dedifferentiation are also associated with declining brain health, such as the accumulation of white matter hyperintensities (Kantarovich et al., 2022). However, unlike healthy aging, our findings in lonely older adults indicate greater within-network connectivity in higher association networks. We previously reported reduced network modularity associated with loneliness in younger adults (Mwilambwe-Tshilobo et al., 2019). While speculative, lonely older adults may compensate for these age and loneliness-related functional changes by increasing within-network connectivity of higher association networks. Future research would benefit from disentangling the combined effects of aging and loneliness on functional brain reorganization.

Finally, our two earlier studies reported divergent associations between loneliness and brain function for younger and middle-aged adults. Interestingly, we only partially replicated the findings from our study involving a sizable middle-aged cohort drawn from the UK Biobank (Spreng et al., 2020). Consistent with Spreng et al. (2020), loneliness in older adults was related to greater RSFC within the default, fronto-parietal control and limbic networks, and associations between the default and limbic network. Unlike Spreng et al. (2020), this pattern extended to greater connectivity within and among ventral and dorsal attention networks in our older adult cohort, indicating that the impact of loneliness on brain function may continue to shift beyond midlife into older age. Our findings provide further evidence that the UK Biobank, representing a large population-based cohort, is a developmentally unique sample (Kiesow et al., 2021) that may not capture brain and behavioral associations observed in early or late adulthood. Future research including an adult life-span sample is needed to fully characterize differences in loneliness and brain associations across the broad continuum of adult human development.

### Shared RSFC Pattern Related to Empathy Across Age Groups

We observed age-invariant associations between subdomains of empathy and RSFC, characterized by greater interactions within the visual network and connections with ventral attention, fronto-parietal control, and default networks. We did not predict this robust age-invariant association given limited evidence relating visual network functioning to empathy (Katsumi et al., 2021; Schurz et al., 2021a, 2021b). We speculate that the dependence of empathic ability on the perception of social cues (Cacioppo & Hawkley, 2009) may underlie the neural patterns observed here.

Few studies have examined the neural correlates of empathy in aging. Decreased activation in the insula and anterior/mid-cingulate (core nodes of the ventral attention network; Chen et al., 2014; Riva et al., 2018) have been associated with affective empathy in older adults. However, recent work failed to find similar age differences (Ziaei et al., 2021). More robust age-related brain differences have been observed for cognitive empathy, specifically implicating the dorsal medial prefrontal cortex, a key node of the default network related to social cognition (Beadle & de la Vega, 2019; Moran et al., 2012). Our observations suggest differing age-related trajectories in empathy and loneliness. The association between empathic functioning and RSFC was age-invariant. In contrast, the association between loneliness and RSFC differed with age, as we report here (Figure 3) and in other age cohorts (e.g., Mwilambwe-Tshilobo et al., 2019; Spreng et al., 2020).

### Differences in RSFC Across Dimensions of Empathic Responding

We included performance-based emotional recognition (RMIE) and self-reported trait empathy measures, allowing us to examine how these different expressions of empathic responding relate to RSFC (Ziaei et al., 2021). As revealed in the second LV (Figure 4), these two aspects of empathic responding are associated with divergent neurocognitive systems, consistent with previous reports that empathic responding encompasses affective and cognitive processes (Christov-Moore et al., 2020; Schurz et al., 2021a, 2021b). Specifically, we identified an age-invariant difference in the relationship between emotion recognition ability using the RMIE task and self-reported trait empathy measures. Younger and older adults shared a common RSFC pattern associated with better performance on the RMIE characterized by greater intra- and internetwork connectivity of association networks. In contrast, self-reported trait empathy measures in younger adults were associated with greater visual network connectivity with the rest of the brain. This divergence in intrinsic network connectivity patterns may reflect functional organizational features of brain networks that enable specialized and flexible social-cognitive functioning. Recent work on brain network interactions related to social-cognitive functioning by Schurz et al. (2020) proposes that differences in network segregation and integration may account for differences in connectivity patterns across theory of mind and empathy tasks. Specifically, interactions between the default network and attention and fronto-parietal networks. However, further evidence is needed to disambiguate differences in network interactions underlying task versus trait-based measures of empathic responding.

### Behavioral Associations Between Loneliness and Empathy

Finally, while not a central aim of the current study, our behavioral findings confirmed previous reports of an inverse association between loneliness and empathy (Nakagawa et al., 2015) observed in younger and older adults (Beadle et al., 2012). While we used a different self-report measure to assess affective and cognitive aspects of empathy (TEQ vs. Empathy Quotient), we also observed a negative association across the entire sample. However, when we examined the relationship between loneliness and subdomains of empathic responding separately in each age group, reliable associations were only observed for older adults on self-report measures assessing the affective features of empathy. This finding may reflect shifts in motivational goals that occur as people age. Socioemotional selectivity theory states that socioemotional goals become salient for older adults (Carstensen, 2006). This change in goal hierarchies in later life shifts cognitive resources toward emotional regulation to meet heightened socioemotional needs (Mather, 2016; Mather & Carstensen, 2005). Thus, the relationship between loneliness and empathy may be heightened in older adulthood, reflecting the importance of maintaining adaptive socioemotional functioning in late-life development. Our results further underscore this point by demonstrating that aspects of relationship quality are important factors to consider when investigating age differences related to loneliness. We show that loneliness was inversely related to the quality of social relationships, and that controlling for social support and friendship attenuated the association between loneliness and RSFC in younger adults, but not older adults.

### Conclusion

Loneliness is a modifiable risk factor associated with various health problems in older adulthood (Ong et al., 2016). Further, the experience of loneliness is negatively related to empathic responding, which is necessary for fostering and maintaining close relationships (Beadle & de la Vega, 2019; Morelli et al., 2017). Here we examined associations between these essential dimensions of sociality, brain function, and differences with age. Our findings revealed that the negative association between loneliness and empathy, observable across the life-span, was greater in older adults. While longitudinal studies are needed to determine causal associations, it is possible that experiencing loneliness in later life may precipitate a cascade of adverse changes in social functioning that could exacerbate feelings of social isolation. We also identified a pattern of age differences in brain function that is differentially related to loneliness and empathy in older, but not younger, adults. Extending our previous work in young (Mwilambwe-Tshilobo et al., 2019) and middle-aged (Spreng et al., 2020) cohorts, the current results demonstrate that loneliness impacts different neurocognitive systems across the adult life-span. Early theoretical accounts implicating loneliness in disordered social perception (Cacioppo & Hawkley, 2009) may not fully capture the experience of loneliness in later life. Lower motivation to form new social bonds and access to a larger store of lived social experiences may shift the impact of loneliness toward more internally directed cognitive processes and associated neural networks, as older adults mentalize and reminiscence to fulfill unmet social desires. Whether and how such a shift may precipitate the adverse cognitive sequelae associated with loneliness in later life is an important direction for future research (Bzdok & Dunbar, 2020, 2022; Spreng & Bzdok, 2021).

## ACKNOWLEDGMENTS

The authors thank Dr. Bratislav Misic for his insight on the PLS analysis.

## SUPPORTING INFORMATION

Supporting information for this article is available at https://doi.org/10.1162/netn_a_00293. All data from the current report are open access and publicly available (see Spreng et al., 2022, for data descriptor). Demographic and behavioral data are available within the Open Science Framework project “Goal-Directed Cognition in Older and Younger Adults” (https://osf.io/yhzxe/); neuroimaging data are available on OpenNeuro (https://openneuro.org/datasets/ds003592).

## AUTHOR CONTRIBUTIONS

Laetitia Mwilambwe-Tshilobo: Conceptualization; Data curation; Formal analysis; Investigation; Methodology; Visualization; Writing – original draft. Roni Setton: Data curation; Writing – review & editing. Danilo Bzdok: Funding acquisition; Writing – review & editing. Gary R. Turner: Funding acquisition; Supervision; Writing – review & editing. R. Nathan Spreng: Conceptualization; Funding acquisition; Supervision; Writing – review & editing.

## FUNDING INFORMATION

R. Nathan Spreng and Danilo Bzdok, National Institutes of Health, Award ID: R01AG068563A. R. Nathan Spreng, Canadian Institute of Health Research.

## TECHNICAL TERMS

Loneliness: The subjective feeling of social isolation when one’s social needs are unmet by the quantity or quality of one’s social relationships.
Empathy: The ability to infer, understand, or simulate the feelings and thoughts of other people.
Neurocognitive systems: Spatially distributed large-scale brain networks associated with cognition.
Internally directed cognitive processes: Mental processes that rely upon attentional allocation to internally generated information (e.g., memory, emotions).
Externally directed cognitive processes: Mental processes that rely upon attentional allocation to extrapersonal space and/or stimuli (e.g., sensory perception).
Multi-echo fMRI: An fMRI acquisition approach that collects multiple echo times (TEs) after each excitation pulse. Based upon the TE dependence of BOLD signal across the brain, multi-echo fMRI can remove signal dropout zones observed with single-echo fMRI, and quantitatively separate BOLD from non-BOLD fMRI signals, to improve image quality.
Group Prior Individual Parcellation (GPIP): An individualized parcellation approach that identifies subject-specific functional neuroanatomy, while preserving parcel labels, across a group of participants. From standardized parcellation schemes, parcel boundaries are tailored to each person’s resting-state functional connectivity data.
Partial least squares (PLS): A multivariate decomposition method that can be used to identify patterns of covariance between a set of brain variables and a set of behaviors.
Latent variables (LVs): In PLS, these are the linear combinations of the input variables that optimally explain orthogonal patterns of covariance.
Brain scores: A PLS output variable that quantifies the extent to which a participant expresses the group connectivity pattern. The value is calculated by taking the dot product of the PLS-derived matrix and the individual subject resting-state functional connectivity matrix.
