# Supplementary material for: Age differences in functional brain networks associated with loneliness and empathy
Source: Netw Neurosci. 2023 Jun 30;7(2):496–521. doi: 10.1162/netn_a_00293 (PMC10312262; doi:10.1162/netn_a_00293)
Supplement: Supplementary file 1 [file netn-7-2-496-s001.pdf]

Mwilambwe-Tshilobo, L., Setton, R., Bzdok, D., Turner, G. R. & Spreng, R. N. (2022). Supporting information for “Age differences in functional brain networks associated with loneliness and empathy”. *Network Neuroscience*. Advance publication. https://doi.org/10.1162/netn_a_00293

Supporting Information for “Age differences in functional brain networks associated with loneliness and empathy”

Supplementary Results

1.1 Behavioral and RSFC associations controlling for quantity and quality of social relationships

There is a possibility that aspects of social relationship quantity and quality may differentially impact the effects of loneliness across age groups. Therefore, we wanted to examine the impact of objective and subjective characteristics of social networks in younger and older adults. Two separate partial correlation analyses were performed using social network size (quantity), and subjective perception of social support and friendship (quality) were included as additional covariates for each LV. For LV1, partial correlations between the PLS brain scores and behavioral measures remained significant when social network size was included as a covariate (top section of Supplementary Table 2B). When measures of relationship quality were included as covariates, all brain-behavior associations remained significant except for the correlation between RSFC and loneliness in younger adults, which was trending (pr = 0.07; top section of Supplementary Table 2C). For LV2, when controlling for social network size, an additional positive brain-behavior correlation emerged for empathic concern in older adults (bottom section of Supplementary Table 2B). When social support and friendship measures were included as covariates, only perspective taking remained significant from the primary PLS results. However, the brain score- RMIE association became significant (bottom section of Supplementary Table 2B).

Mwilambwe-Tshilobo, L., Setton, R., Bzdok, D., Turner, G. R. & Spreng, R. N. (2022). Supporting information for “Age differences in functional brain networks associated with loneliness and empathy”. *Network Neuroscience*. Advance publication. https://doi.org/10.1162/netn_a_00294

Supplementary Table 1.

Correlation of UCLA loneliness with empathic responding measures in younger and older adults

Table 1A

Empathy Measure	Younger Adults *(n=128)*	Older Adults *(n=92)*	Full Sample *(n=220)*
RMIE	0.05 (0.60) [-0.12, 0.22]	0.07 (0.5) [-0.13, 0.27]	0.07 (0.41) [-0.09, 0.22]
TEQ	-0.03 (0.67) [-0.12, 0.14]	**-0.27 (0.009)* [-0.45, -0.07]**	-0.12 (0.14) [-0.27, 0.04]
Perspective Taking	-0.07 (0.44) [-0.24, 0.11]	**-0.31 (0.003)* [-0.48, -0.11]**	**-0.16 (0.04)* [-0.31, -0.01]**
Empathic Concern	-0.03 (0.73) [-0.2, 0.14]	**-0.33 (0.001)* [-0.5, -0.14]**	**-0.20 (0.01)* [-0.34, -0.05]**

Table 1B

	Younger Adults *(n= 118)*	Older Adults *(n=87)*	Full Sample *(n=205)*
RMIE	0.10 (0.30) [-0.09, 0.28]	0.10 (0.39) [-0.12, 0.31]	0.08 (0.25) [-0.6, 0.22]
TEQ	-0.08 (0.40) [-0.26, 0.11]	**-0.22 (0.05)* [-0.42, -0.0]**	**-0.15 (0.04)* [-0.28, -0.01]**
Perspective Taking	0.04 (0.65) [-0.14, 0.23]	-0.22 (0.05) [-0.48, 0.0]	-0.05 (0.46) [-0.19, 0.09]
Empathic Concern	-0.03 (0.73) [-0.22, 0.15]	**-0.22 (0.05)* [-0.42, -0.0]**	-0.09 (0.19) [-0.23, 0.05]

Table 1C

	Younger Adults *(n= 89)*	Older Adults *(n=70)*	Full Sample *(n=159)*
RMIE	0.05 (0.65) [-0.17, 0.26]	0.08 (0.55) [-0.17, 0.32]	0.06 (0.46) [-0.1, 0.22]
TEQ	-0.03 (0.82) [-0.24, 0.19]	-0.17 (0.19) [-0.39, 0.08]	-0.09 (0.25) [-0.25, 0.07]
Perspective Taking	0.16 (0.14) [-0.05, 0.37]	-0.18 (0.17) [-0.4, 0.07]	0.03 (0.72) [-0.13, 0.19]
Empathic Concern	0.04 (0.74) [-0.18, 0.25]	-0.19 (0.13) [-0.42, -0.06]	-0.05 (0.58) [-0.2, 0.11]

Table 1D

	Younger Adults *(n= 117)*	Older Adults *(n=86)*	Full Sample *(n=203)*
RMIE	0.15 (0.13) [-0.04, 0.33]	0.22 (0.05) [-0.0, 0.42]	**0.15 (0.04) [0.1, 0.28]**
TEQ	-0.01 (0.94) [-0.2, 0.18]	-0.17 (0.13) [-0.38, 0.05]	-0.10 (0.17) [-0.24, 0.04]
Perspective Taking	0.11 (0.27) [-0.08, 0.29]	-0.08 (0.50) [-0.29, 0.15]	0.05 (0.49) [-0.09, 0.19]
Empathic Concern	-0.12 (0.20) [-0.3, 0.07]	-0.07 (0.55) [-0.29, 0.16]	-0.07 (0.31) [-0.21, 0.07]

Supplementary Table 1 Note: Correlation and partial correlation values between UCLA loneliness and empathic functioning in younger and older adults. *p*-values are in parentheses and 95% confidence intervals in square brackets. Table 1A: Full product-moment correlations within the younger and older adult cohorts. Table 1B: Relationship between UCLA loneliness and

empathic responding, controlling for gender, site, education, neuroticism, and cognitive composite score. Table 1C: Relationship between UCLA loneliness and empathic responding, controlling for gender, site, education, neuroticism, cognitive composite score and social network size. Table 1D: Relationships between UCLA loneliness and empathic responding controlling for gender, site, education, neuroticism, cognitive composite score, instrumental support, emotional support, and friendship. For the full sample in all Tables 1B-D, age is controlled for. Note the lower sample size with increasing number of covariates due to missing data. TEQ= Toronto Empathy Questionnaire; RMIE= Reading the Mind in the Eyes Task. * $p < 0.05$; ** $p < 0.01$. *** $p < 0.001$

Supplementary Table 2A.

Correlation between PLS brain scores and behavioral measures with covariates

Group	Behavior	n	r	CI95%	*p*-value [1]
			LV 1		
Younger Adults	Loneliness	118	0.20	[0.01, 0.37]	**0.04**
Younger Adults	RMIE	118	0.14	[-0.04, 0.32]	0.13
Younger Adults	TEQ	118	0.36	[0.19, 0.51]	**<0.001**
Younger Adults	PT	118	0.33	[0.15, 0.49]	**<0.001**
Younger Adults	EC	118	0.26	[0.08, 0.43]	0.01
Older Adults	Loneliness	87	-0.32	[-0.51, -0.11]	**<0.001**
Older Adults	RMIE	87	0.14	[-0.08, 0.35]	0.21
Older Adults	TEQ	87	0.63	[0.47, 0.74]	**<0.001**
Older Adults	PT	87	0.66	[0.52, 0.77]	**<0.001**
Older Adults	EC	87	0.77	[0.67, 0.85]	**<0.001**
			LV2		
Younger Adults	Loneliness	118	-0.11	[-0.29, 0.07]	0.23
Younger Adults	RMIE	118	0.06	[-0.13, 0.24]	0.55
Younger Adults	TEQ	118	-0.56	[-0.68, -0.42]	**<0.001**
Younger Adults	PT	118	-0.55	[-0.67, -0.41]	**<0.001**
Younger Adults	EC	118	-0.51	[-0.63, -0.36]	**<0.001**
Older Adults	Loneliness	87	-0.09	[-0.3, 0.13]	0.42
Older Adults	RMIE	87	0.48	[0.29, 0.63]	**<0.001**
Older Adults	TEQ	87	-0.01	[-0.22, 0.21]	0.96
Older Adults	PT	87	0.37	[0.17, 0.55]	**<0.001**
Older Adults	EC	87	0.15	[-0.07, 0.36]	0.17

Supplementary Table 2A Note. Covariates include age, gender, site, education, neuroticism, and cognition composite score. Participants with missing data on one more of the covariates were omitted from the brain-behavior correlation analysis. TEQ= Toronto Empathy Questionnaire; RMIE= Reading the Mind in the Eyes Task; PT = Perspective Taking; EC= Empathic Concern.

[1] To account for the possibility that neuroticism may confound the age effects observed in our PLS analysis we ran the correlations excluding neuroticism. The results shown here remained consistent.

Mwilambwe-Tshilobo, L., Setton, R., Bzdok, D., Turner, G. R. & Spreng, R. N. (2022). Supporting information for “Age differences in functional brain networks associated with loneliness and empathy”. *Network Neuroscience*. Advance publication. https://doi.org/10.1162/netn_a_00294

Mwilambwe-Tshilobo, L., Setton, R., Bzdok, D., Turner, G. R. & Spreng, R. N. (2022). Supporting information for "Age differences in functional brain networks associated with loneliness and empathy". *Network Neuroscience*. Advance publication. https://doi.org/10.1162/netn_a_00294

Supplementary Table 2B.

Correlation between PLS brain scores and behavioral measures with covariates (including social network size)

Group	Behavior	n	r	CI95%	*p*-value[2]
			LV 1		
Younger Adults	Loneliness	89	0.28	[0.07, 0.47]	**0.01**
Younger Adults	RMIE	89	0.19	[-0.03, 0.39]	0.09
Younger Adults	TEQ	89	0.25	[0.04, 0.45]	**0.02**
Younger Adults	PT	89	0.34	[0.13, 0.52]	**<0.001**
Younger Adults	EC	89	0.19	[-0.03, 0.39]	0.09
Older Adults	Loneliness	70	-0.30	[-0.51, -0.06]	**0.02**
Older Adults	RMIE	70	0.19	[-0.06, 0.42]	0.14
Older Adults	TEQ	70	0.59	[0.40, 0.73]	**<0.001**
Older Adults	PT	70	0.68	[0.52, 0.79]	**<0.001**
Older Adults	EC	70	0.77	[0.64, 0.85]	**<0.001**
			LV2		
Younger Adults	Loneliness	89	-0.19	[-0.39, 0.03]	0.08
Younger Adults	RMIE	89	0.04	[-0.18, 0.26]	0.72
Younger Adults	TEQ	89	-0.58	[-0.71, -0.41]	**<0.001**
Younger Adults	PT	89	-0.54	[-0.68, -0.37]	**<0.001**
Younger Adults	EC	89	-0.47	[-0.63, -0.29]	**<0.001**
Older Adults	Loneliness	70	-0.13	[-0.37, 0.12]	0.30
Older Adults	RMIE	70	0.51	[0.30, 0.67]	<**0.001**
Older Adults	TEQ	70	0.11	[-0.14, 0.35]	0.39
Older Adults	PT	70	0.44	[0.22, 0.62]	**<0.001**
Older Adults	EC	70	0.34	[0.10, 0.54]	**0.01**

Supplementary Table 2B Note. Covariates include age, gender, site, education, neuroticism, cognition composite score, and social network size. TEQ= Toronto Empathy Questionnaire; RMIE= Reading the Mind in the Eyes Task; PT = Perspective Taking; EC= Empathic Concern.

[2] Results remained significant when excluding neuroticism demonstrating that the age effects are not confounded by neuroticism.

Mwilambwe-Tshilobo, L., Setton, R., Bzdok, D., Turner, G. R. & Spreng, R. N. (2022). Supporting information for “Age differences in functional brain networks associated with loneliness and empathy”. *Network Neuroscience*. Advance publication. https://doi.org/10.1162/netn_a_00294

Supplementary Table 2C.

Correlation between PLS brain scores and behavioral measures with covariates (including subjective perception for quality of social relationships)

Group	Behavior	n	r	CI95%	*p*-value
			LV 1		
Younger Adults	Loneliness	117	0.18	[-0.01, 0.35]	0.07
Younger Adults	RMIE	117	0.14	[-0.05, 0.32]	0.16
Younger Adults	TEQ	117	0.38	[0.20, 0.53]	**<0.001**
Younger Adults	PT	117	0.34	[0.16, 0.49]	**<0.001**
Younger Adults	EC	117	0.26	[0.07, 0.43]	**0.01**
Older Adults	Loneliness	86	-0.25	[-0.45, -0.03]	**0.03**
Older Adults	RMIE	86	0.10	[-0.13, 0.32]	0.39
Older Adults	TEQ	86	0.62	[0.46, 0.74]	**<0.001**
Older Adults	PT	86	0.65	[0.50, 0.76]	**<0.001**
Older Adults	EC	86	0.76	[0.65, 0.84]	**<0.001**
			LV2		
Younger Adults	Loneliness	117	-0.17	[-0.35, 0.02]	0.07
Younger Adults	RMIE	117	0.08	[-0.11, 0.26]	0.43
Younger Adults	TEQ	117	-0.56	[-0.68, -0.42]	**<0.001**
Younger Adults	PT	117	-0.55	[-0.67, -0.40]	**<0.001**
Younger Adults	EC	117	-0.52	[-0.64, -0.37]	**<0.001**
Older Adults	Loneliness	86	0.02	[-0.21, 0.24]	0.88
Older Adults	RMIE	86	0.46	[0.26, 0.62]	**<0.001**
Older Adults	TEQ	86	-0.04	[-0.26, 0.19]	0.73
Older Adults	PT	86	0.33	[0.11, 0.51]	**<0.001**
Older Adults	EC	86	0.11	[-0.11, 0.33]	0.32

Supplementary Table 2C Note. Impact of relationship quality on RSFC-behavior associations. Covariates include age, gender, site, education, neuroticism, cognition composite score, emotional support, instrumental support, and friendship. The last three covariates were included as measures of participants' subjective perception of the social support and companionship available to them. The PLS results remain significant in both LVs except for the correlation between PLS brain scores and loneliness in younger adults (LV1). TEQ= Toronto Empathy Questionnaire; RMIE= Reading the Mind in the Eyes Task; PT = Perspective Taking; EC= Empathic Concern.

1.2 Characterizing associations between RSFC, loneliness, and empathic functioning

The RSFC data used in the primary PLS results focused on six networks: visual, dorsal attention, ventral attention, limbic, frontoparietal control, and default. To provide a comprehensive assessment of the PLS results using whole-brain RSFC, we conducted a subsequent analysis that included the somatosensory network. Although the patterns identified were comparable to that of the initial PLS results, only LV2 remained significant (LV1: $p = 0.12$; 25.06% covariance explained; LV2: $p = 0.01$; 16.22% covariance explained).

Supplementary Figure 1.

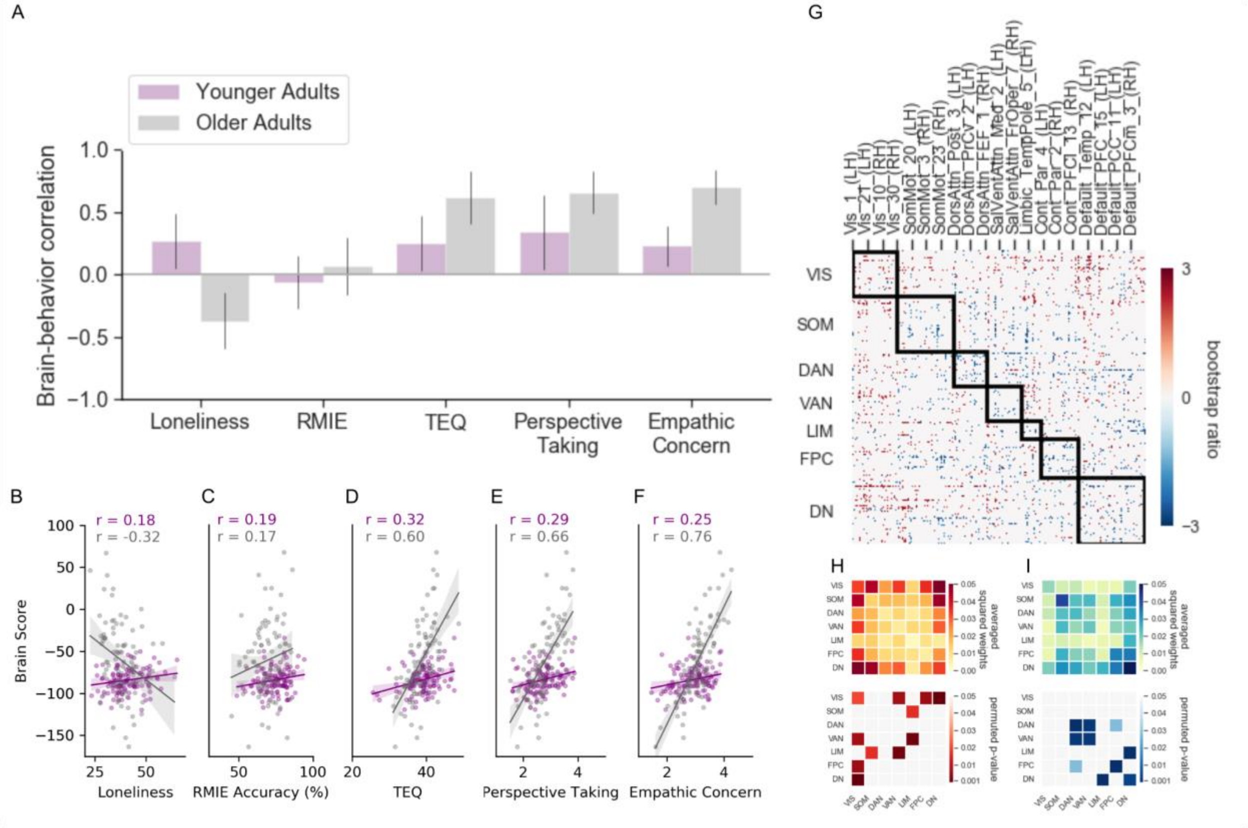

Supplementary Figure 1. PLS analysis of covariance between whole-brain and behavior for LV1. (A) Displays the correlation between behavioral loneliness, empathic responding measures, and functional connectivity in younger and older adults. Error bars show 95% confidence intervals

determined by bootstrap through bootstrap resampling. Scatterplots in panels B-F show participant brain scores corrected for age, site, gender, education, neuroticism, and cognition as a function of each behavioral measure. Whole-brain functional networks are based on the Yeo 7-network solution. (G) Correlation matrix of the reliable pairwise functional connections associated with behavior. The matrix bootstrap ratios are thresholded at ± 2 to 3. Network-level contributions to the positive (H) and negative (I) connectivity pattern for LV1: Top matrices show the averaged squared salience weights, which reflects a summary of the connectivity pattern; bottom matrices show significant network contribution estimated using permutation testing on the correlation matrix in (G). Behaviors that correlate positively with the pattern are represented in warm colors and negative brain-behavior correlations in cool colors. VIS = visual, SOM = somatomotor, DAN = dorsal attention, VAN = ventral attention, LIM = limbic, FPC = frontoparietal control, DN = default.

Supplementary Figure 2.

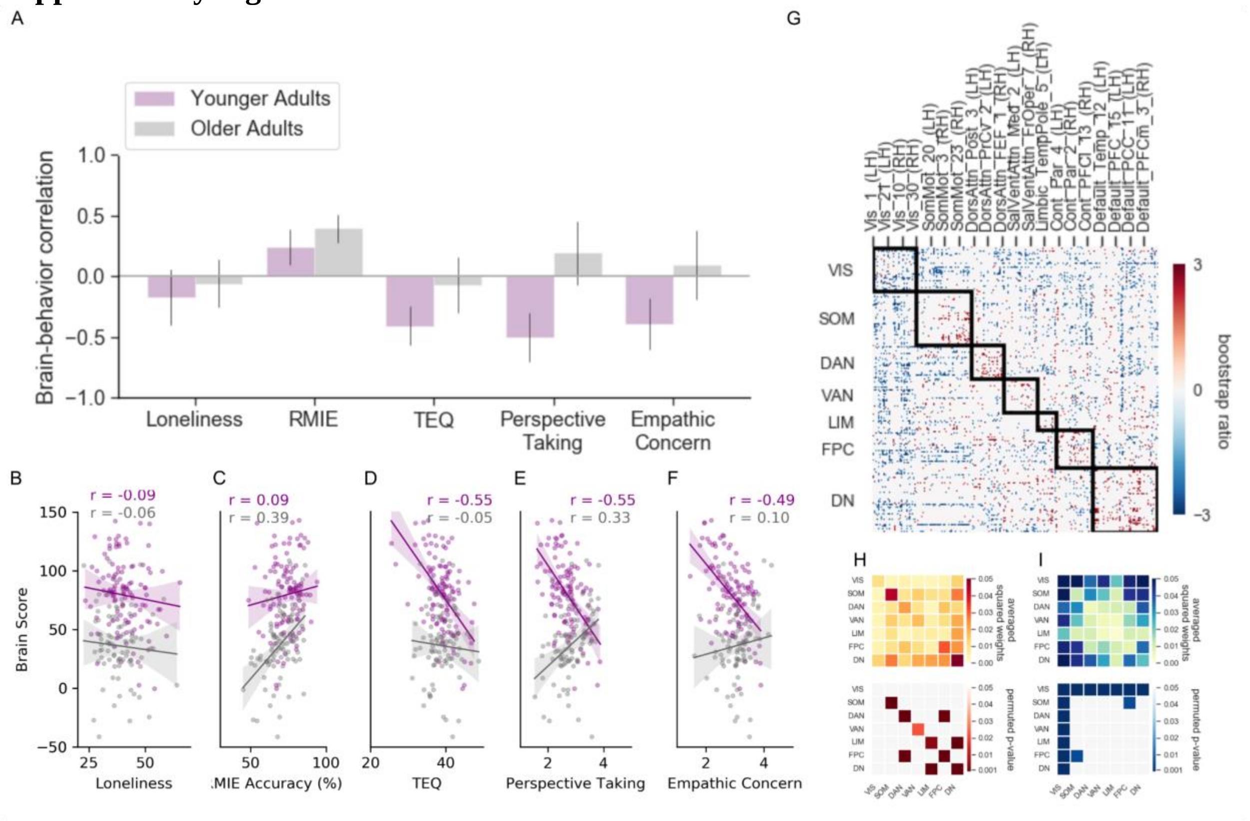

Supplementary Figure 2. PLS analysis of covariance between whole-brain and behavior for LV2. (A) Displays the correlation between behavioral loneliness, empathy responding, and functional connectivity in younger and older adults. Error bars show 95% confidence intervals determined by bootstrap through bootstrap resampling. Scatterplots in panels B-F show participant brain scores corrected for age, site, gender, education, neuroticism, and cognition as a function of each behavioral measure. Whole-brain functional networks are based on the Yeo 7-network solution. (G) Correlation matrix of the reliable pairwise functional connections associated with behavior.

Mwilambwe-Tshilobo, L., Setton, R., Bzdok, D., Turner, G. R. & Spreng, R. N. (2022). Supporting information for “Age differences in functional brain networks associated with loneliness and empathy”. *Network Neuroscience*. Advance publication. https://doi.org/10.1162/netn_a_00294

The matrix bootstrap ratios are thresholded at ± 2 to 3. Network-level contributions to the positive (H) and negative (I) connectivity pattern for LV2: Top matrices show the averaged squared salience weights, which reflects a summary of the connectivity pattern; bottom matrices show significant network contribution estimated using permutation testing on the correlation matrix in (G). Behaviors that correlate positively with the pattern are represented in warm colors and negative brain-behavior correlations in cool colors. VIS = visual, SOM = somatomotor, DAN = dorsal attention, VAN = ventral attention, LIM = limbic, FPC = frontoparietal control, DN = default.

Supplementary Figure 3.

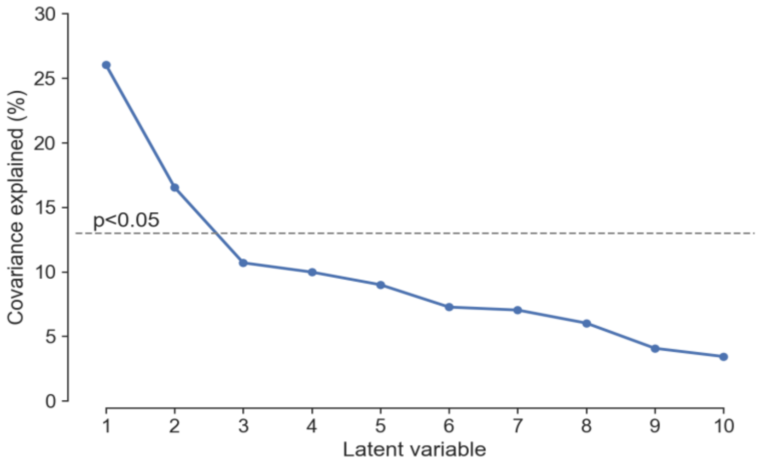

Supplementary Figure 3. Scree plot of covariance explained for each latent variable from the partial least-squares (PLS) analysis. The first latent variable explains 26.02% of the variance, and the second latent variable explains 16.53%. Latent variables with covariance values above the dotted line significantly captured the relationship between loneliness and aspects of empathic responding in younger and older adults.

Supplementary Figure 4.

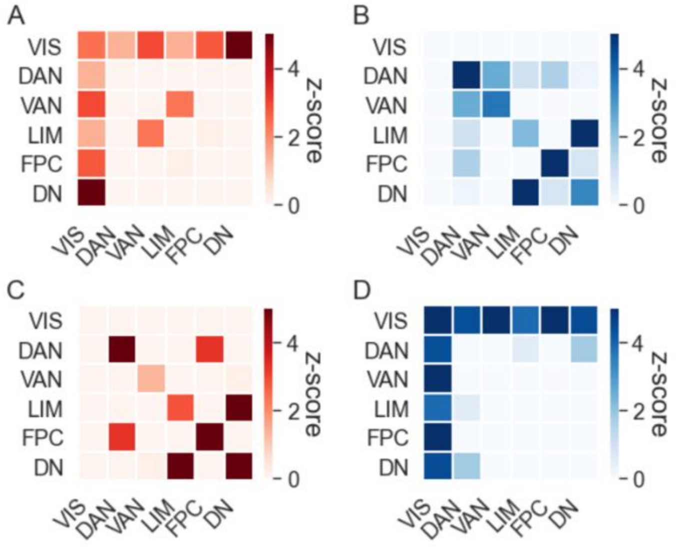

Supplementary Figure 4. The primary PLS analysis's network contributions for LV1 and LV2 are expressed as z-scores relative to the permuted null model. The mean contribution is computed for all intra- and inter-network connections. The top row shows the mean (A) positive and (B) negative RSFC pattern for LV1. The matrices in the bottom row are the mean (C) positive and (D) negative RSFC patterns for LV2. Higher values indicate greater than-expected contributions of the network pairs to the respective PLS-derived RSFC pattern for LV1(Figure 3G) and LV2 (Figure 4G).

1.3 Impact of Age on RSFC. The PLS results captured age-dependent differences in the association between loneliness and RSFC for LV1 and an age-independent association between subdomains of empathy and RSFC. Although we control for age in the partial correlations between RSFC and behaviors, we wanted to rule out the possibility that this result was not due to age loading strongly onto LV1. To assess this, age was partialled out from the original X (RSFC) and Y (behavior) matrices and repeated the PLS analysis. Similar to the initial analysis, LV1 was significant and captured an age difference in RSFC related to loneliness between younger and older adults (LV1: $p = 0.03$; explained 25.5% of the covariance; Supplementary Figure 5). LV2 was also significant and captured an age-independent association between RSFC and empathy (LV2: $p = 0.006$; 17.92 % covariance explained; Supplementary Figure 6).

Brain-behavior correlations remained largely unchanged for both LVs. The most notable differences from the original results emerged for LV1 at the network level (Supplementary Figure 5 H-I). The positive expression of LV1 was driven solely by the connectivity of the visual

network, and the negative expression of LV included an additional network interaction between the frontoparietal and dorsal attention networks. The relationship between loneliness and RSFC was still age-independent; however, unlike in the primary analysis, the correlation for older adults was reliable. Supplementary Figure 7 shows the covariance of LV1 and LV2 before and after partialling age from the X and Y matrices. If X and Y were dependent on age, we might see a significant decrease in the effect size for LV1 after partialling out age. We did not observe this, suggesting that our primary for LV2 is not due to age strongly loading to LV1.

Supplementary Figure 5.

Mwilambwe-Tshilobo, L., Setton, R., Bzdok, D., Turner, G. R. & Spreng, R. N. (2022). Supporting information for “Age differences in functional brain networks associated with loneliness and empathy”. *Network Neuroscience*. Advance publication. https://doi.org/10.1162/netn_a_00294

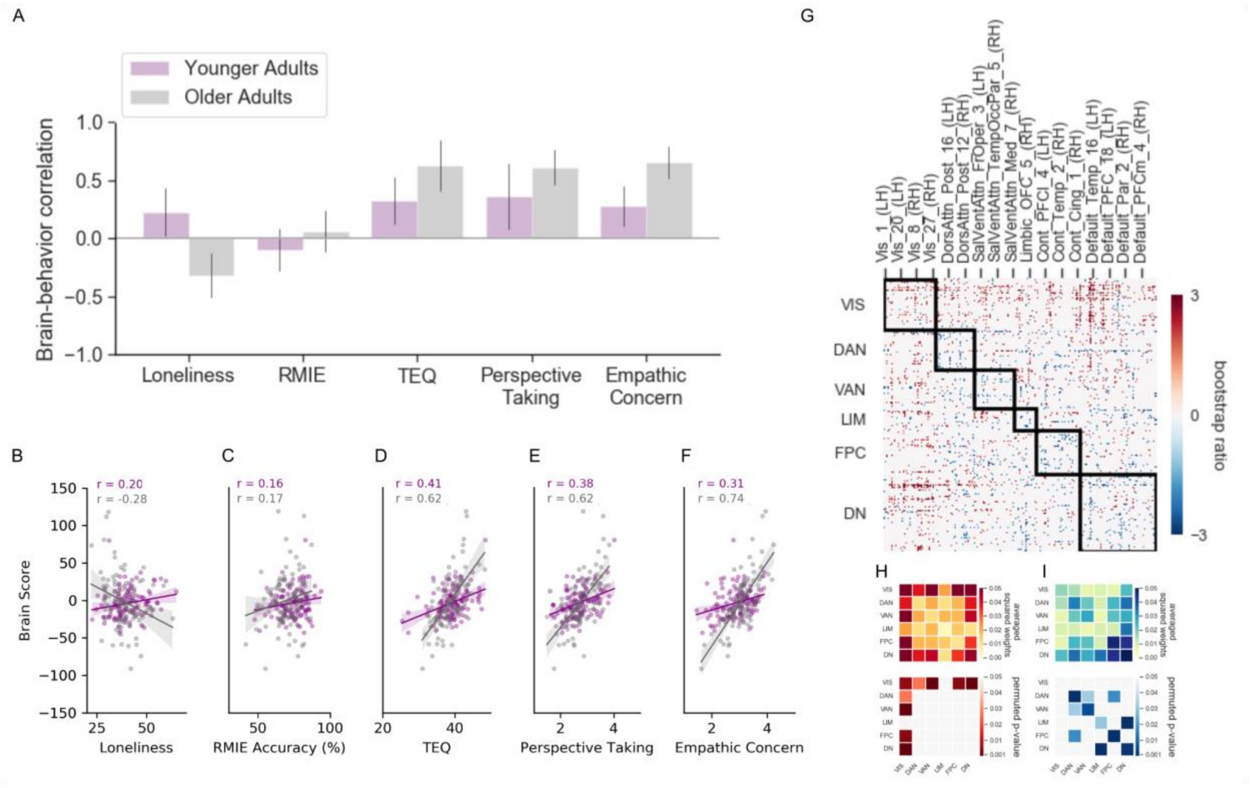

Supplementary Figure 5. PLS analysis of covariance between RSFC and behavior controlling for age for LV1. The covariance pattern captured by LV1 was significant (p = 0.03) and accounted for 25.5% of the covariance. (A) Displays the correlation between behavioral measures (loneliness, emotional recognition, and empathic ability) and functional connectivity in younger and older adults. Error bars show 95% confidence intervals determined by bootstrap resampling. Scatterplots in panels B-F show correlations between participant brain scores and behavioral measures controlling for site, gender, education, neuroticism, and cognition. (G) Correlation matrix of the reliable pairwise functional connections associated with behavior. The matrix bootstrap ratios are thresholded at ± 2 to 3. Network-level contributions to the positive (H) and negative (I) connectivity pattern for LV1: Top matrices show the averaged squared salience weights, which reflects a summary of the connectivity pattern; bottom matrices show significant network contribution estimated using permutation testing on the correlation matrix in (G). Behaviors that correlate positively with the pattern are represented in warm colors and negative brain-behavior correlations in cool colors. VIS = visual, DAN = dorsal attention, VAN = ventral attention, LIM = limbic, FPC = frontoparietal control, DN = default.

Mwilambwe-Tshilobo, L., Setton, R., Bzdok, D., Turner, G. R. & Spreng, R. N. (2022). Supporting information for “Age differences in functional brain networks associated with loneliness and empathy”. *Network Neuroscience*. Advance publication. https://doi.org/10.1162/netn_a_00294

Supplementary Figure 6.

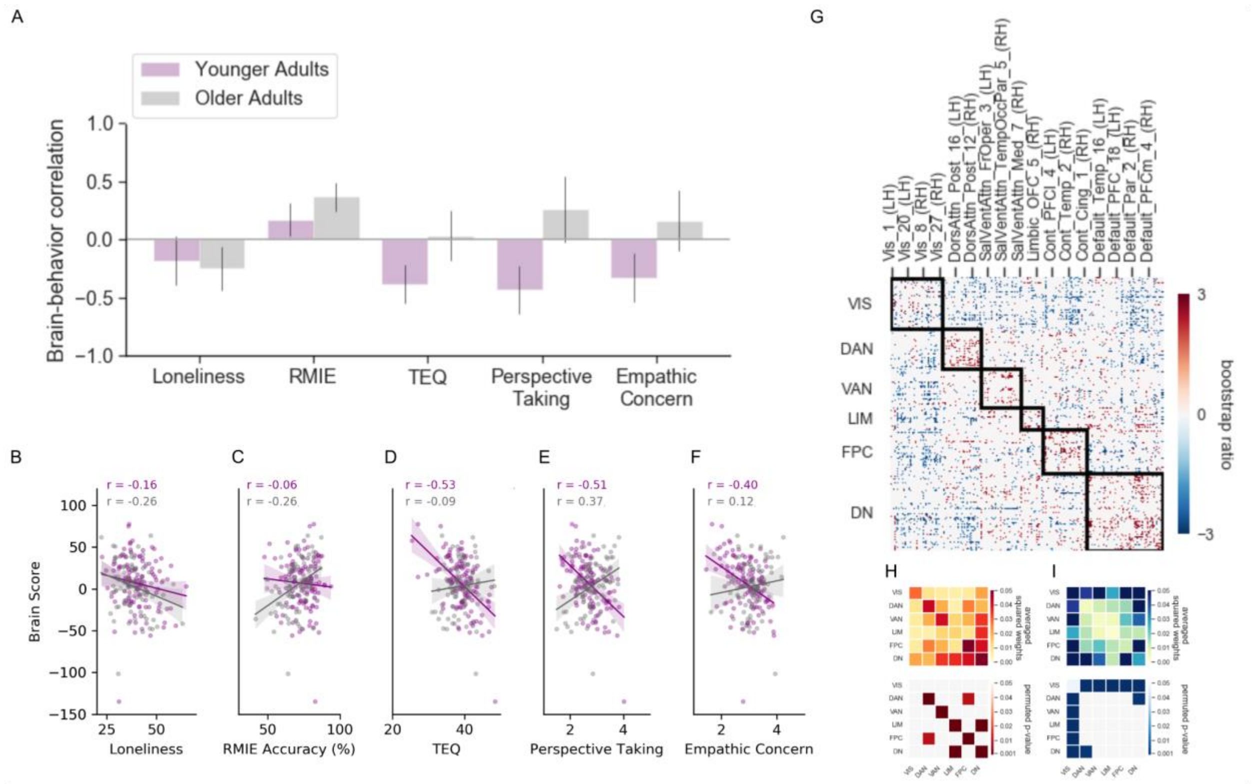

Supplementary Figure 6. PLS analysis of covariance between RSFC and behavior controlling for age in LV2. The covariance pattern captured by LV2 was significant (p = 0.006) and accounted for 17.92% of the covariance. (A) Displays the correlation between behavioral measures (loneliness, affective theory of mind, and empathic ability) and functional connectivity in younger and older adults. Error bars show 95% confidence intervals determined by bootstrap resampling. Scatterplots in panels B-F show correlations between participant brain scores and behavioral measures controlling for site, gender, education, neuroticism, and cognition. (G) Correlation matrix of the reliable pairwise functional connections associated with behavior. The matrix bootstrap ratios are thresholded at ± 2 to 3. Network-level contributions to the positive (H) and negative (I) connectivity pattern for LV2: Top matrices show the averaged squared salience weights, which reflects a summary of the connectivity pattern; bottom matrices show significant network contribution estimated using permutation testing on the correlation matrix in (G). Behaviors that correlate positively with the pattern are represented in warm colors and negative brain-behavior correlations in cool colors. VIS = visual, DAN = dorsal attention, VAN = ventral attention, LIM = limbic, FPC = frontoparietal control, DN = default.

Mwilambwe-Tshilobo, L., Setton, R., Bzdok, D., Turner, G. R. & Spreng, R. N. (2022). Supporting information for "Age differences in functional brain networks associated with loneliness and empathy". *Network Neuroscience*. Advance publication. https://doi.org/10.1162/netn_a_00294

Supplementary Figure 7.

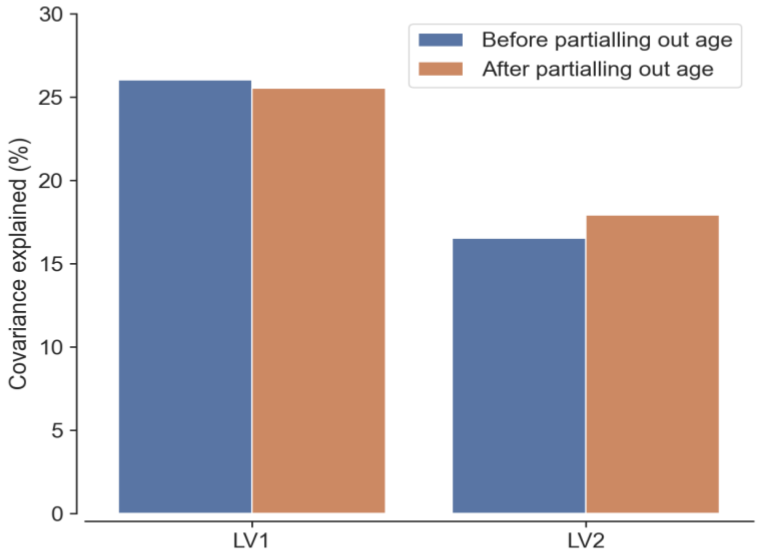

Supplementary Figure 7. Comparison of latent variable covariance before and after partialling out age from the PLS input matrices.

1.4 Impact of motion on RSFC. The participants included in the present study were part of a large cohort (n = 301), for which analyses related to the impact of motion on RSFC indicate residual motion effects (Setton & Mwilambwe-Tshilobo et al., 2022). Using mean FD calculated from the middle-echo (Tz_2; before processing), they reported no age differences or interactions with motion when assessing the RSFC data of younger and older adults. To account for the possibility that residual motion effects did not confound the main findings in the current subset of participants, we first conducted an ANOVA to test for age group and site differences in FD, controlling for site, gender, education, neuroticism, and cognition (Supplementary Table 3). Results found a main effect of age group ($F(1,216)=13.28$, $p < .001$, $\eta p2=.06$) and site ($F(1,216)=1.39$ $p = .24$, $\eta p2= .01$), but no interaction ($F(1,216)=1.52$, $p = .22$, $\eta p2= .01$). Follow up t-tests revealed that older adults had higher FD than younger adults ($T(219)= -39.89$, $p < .001$, [-1.35, -1.24], Cohen's d = 3.7).

Next, we conducted a PLS analysis examining the association between RSFC and FD. Similar to the results reported in the larger cohort from which participants were drawn (Setton &

Mwilambwe-Tshilobo et al., 2022), a significant pattern emerged representing the main effect of motion (LV1: p= 0.02; 57.98% covariance explained; younger adults r=0.84; older adults r =0.82). In addition, we found no significant age group or interactions (LV2: p= 0.47; 42.07% covariance explained), indicating that motion was not a confound in the age group differences. Using the PLS brain scores, we further confirmed that the RSFC pattern that covaried with FD was not associated with age differences in RSFC by performing a partial correlation controlling for gender, education, cognition, and site. Partial correlations between whole-brain RSFC PLS brain scores and FD PLS brain scores were not significant (Supplementary Figure 8).

Finally, to account for the possibility of residual motion impacting the primary PLS results with loneliness and empathic functioning, we included mean FD as a covariate in all analyses, as reported in the main text (Supplementary Table 4). All results held, suggesting they were robust to any residual motion in the data.

Supplementary Table 3.
ANOVA results for age group and site differences on framewise displacement

Predictor	Sum of Squares	df	Mean Squares	F	p	Partial η^2
Group	0.04	1	0.04	13.28	<0.001	0.06
Site	0.04	1	0.004	1.39	0.24	0.01
Group x Site	0.04	1	0.004	1.52	0.22	0.01
Error	0.61	216	0.003			

Supplementary Table 3 Note. Partial η^2 indicates partial eta-squared

Mwilambwe-Tshilobo, L., Setton, R., Bzdok, D., Turner, G. R. & Spreng, R. N. (2022). Supporting information for "Age differences in functional brain networks associated with loneliness and empathy". *Network Neuroscience*. Advance publication. https://doi.org/10.1162/netn_a_00294

Supplementary Figure 8.

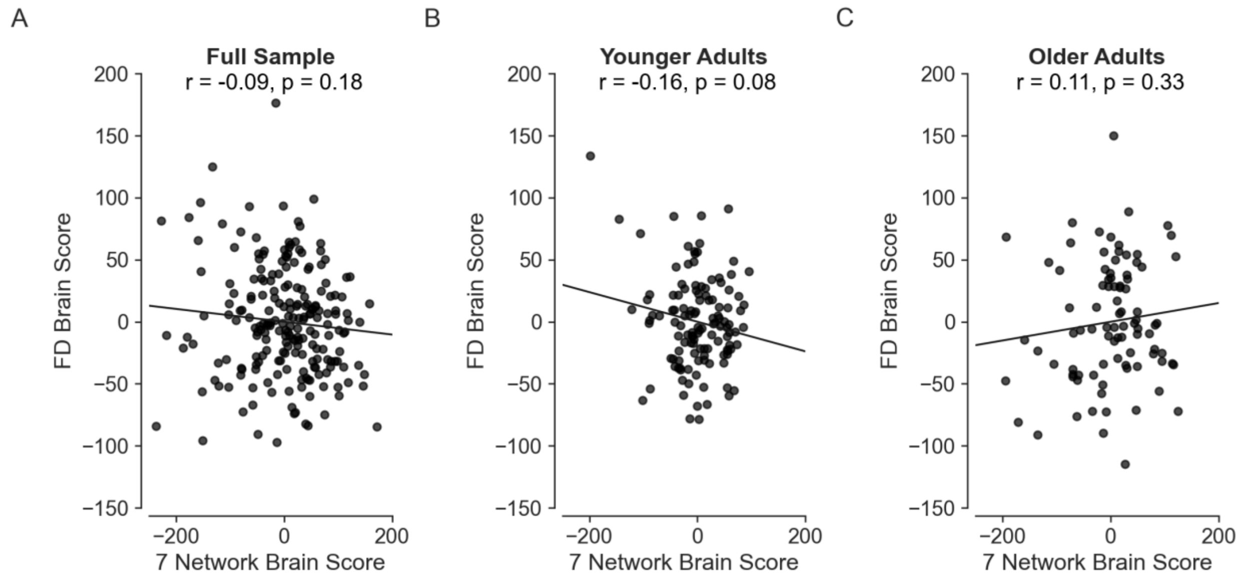

Supplementary Figure 8. No relationship between whole-brain PLS brain scores and motion-related PLS brain scores. Framewise displacement (FD) was calculated on the middle echo (TE_2) prior to processing. Scatterplots show age differences in whole-brain PLS brain scores on the x-axis and motion-related PLS brain scores on the y-axis (from the six networks included in the primary results) across the (A) full sample, (B) in younger adults, and (C) in older adults.

Mwilambwe-Tshilobo, L., Setton, R., Bzdok, D., Turner, G. R. & Spreng, R. N. (2022). Supporting information for “Age differences in functional brain networks associated with loneliness and empathy”. *Network Neuroscience*. Advance publication. https://doi.org/10.1162/netn_a_00294

Supplementary Table 4.
Correlation between PLS brain scores and behavioral measures controlling for covariates (including post-processing mean framewise displacement)

Group	Behavior	n	r	CI95%	p-value
			LV 1		
Younger Adults	Loneliness	118	0.20	[0.01, 0.37]	**0.03**
Younger Adults	RMIE	118	0.14	[-0.05, 0.32]	0.14
Younger Adults	TEQ	118	0.36	[0.19, 0.51]	**<0.001**
Younger Adults	PT	118	0.33	[0.15, 0.49]	**<0.001**
Younger Adults	EC	118	0.26	[0.08, 0.43]	**0.005**
Older Adults	Loneliness	87	-0.32	[-0.51, -0.11]	**0.003**
Older Adults	RMIE	87	0.14	[-0.08, 0.35]	0.21
Older Adults	TEQ	87	0.63	[0.47, 0.74]	**<0.001**
Older Adults	PT	87	0.67	[0.57, 0.77]	**<0.001**
Older Adults	EC	87	0.77	[0.67, 0.85]	**<0.001**
			LV2		
Younger Adults	Loneliness	118	-0.11	[-0.29, 0.08]	0.26
Younger Adults	RMIE	118	0.06	[-0.13, 0.24]	0.54
Younger Adults	TEQ	118	-0.56	[-0.68, -0.42]	**<0.001**
Younger Adults	PT	118	-0.55	[-0.67, -0.40]	**<0.001**
Younger Adults	EC	118	-0.51	[-0.63, -0.36]	**<0.001**
Older Adults	Loneliness	87	-0.09	[-0.31, 0.13]	0.42
Older Adults	RMIE	87	0.48	[0.29, 0.63]	**<0.001**
Older Adults	TEQ	87	-0.005	[-0.22, 0.21]	0.96
Older Adults	PT	87	0.37	[0.17, 0.55]	**0.001**
Older Adults	EC	87	0.15	[-0.07, 0.36]	0.17

Supplementary Table 4 Note. Impact of residual motion on RSFC-behavior associations. Covariates include age, gender, site, education, neuroticism, cognition composite score, and post-processing mean FD. TEQ= Toronto Empathy Questionnaire; RMIE= Reading the Mind in the Eyes Task; PT = Perspective Taking; EC= Empathic Concern.
